# β-Globin LCR and Intron Elements Cooperate and Direct Spatial Reorganization for Gene Therapy

**DOI:** 10.1371/journal.pgen.1000051

**Published:** 2008-04-18

**Authors:** Alla Buzina, Mandy Y. M. Lo, Angela Moffett, Akitsu Hotta, Eden Fussner, Rikki R. Bharadwaj, Peter Pasceri, J. Victor Garcia-Martinez, David P. Bazett-Jones, James Ellis

**Affiliations:** 1 Developmental and Stem Cell Biology Program, SickKids, Toronto, Ontario, Canada; 2 Department of Molecular Genetics, University of Toronto, Toronto, Ontario, Canada; 3 Program in Genetics and Genome Biology, SickKids, Toronto, Ontario, Canada; 4 Department of Biochemistry, University of Toronto, Toronto, Ontario, Canada; 5 Department of Internal Medicine, Division of Infectious Diseases, University of Texas Southwestern Medical Center, Dallas, Texas, United States of America; MRC Human Genetics Unit, United Kingdom

## Abstract

The Locus Control Region (LCR) requires intronic elements within β-globin transgenes to direct high level expression at all ectopic integration sites. However, these essential intronic elements cannot be transmitted through retrovirus vectors and their deletion may compromise the therapeutic potential for gene therapy. Here, we systematically regenerate functional β-globin intron 2 elements that rescue LCR activity directed by 5′HS3. Evaluation in transgenic mice demonstrates that an Oct-1 binding site and an enhancer in the intron cooperate to increase expression levels from LCR globin transgenes. Replacement of the intronic AT-rich region with the Igμ 3′MAR rescues LCR activity in single copy transgenic mice. Importantly, a combination of the Oct-1 site, Igμ 3′MAR and intronic enhancer in the BGT158 cassette directs more consistent levels of expression in transgenic mice. By introducing intron-modified transgenes into the same genomic integration site in erythroid cells, we show that BGT158 has the greatest transcriptional induction. 3D DNA FISH establishes that induction stimulates this small 5′HS3 containing transgene and the endogenous locus to spatially reorganize towards more central locations in erythroid nuclei. Electron Spectroscopic Imaging (ESI) of chromatin fibers demonstrates that ultrastructural heterochromatin is primarily perinuclear and does not reorganize. Finally, we transmit intron-modified globin transgenes through insulated self-inactivating (SIN) lentivirus vectors into erythroid cells. We show efficient transfer and robust mRNA and protein expression by the BGT158 vector, and virus titer improvements mediated by the modified intron 2 in the presence of an LCR cassette composed of 5′HS2-4. Our results have important implications for the mechanism of LCR activity at ectopic integration sites. The modified transgenes are the first to transfer intronic elements that potentiate LCR activity and are designed to facilitate correction of hemoglobinopathies using single copy vectors.

## Author Summary

Expression of the β-globin gene is regulated by interactions between a distant Locus Control Region (LCR) and regulatory elements in or near the gene. We previously showed that LCR activity requires specific β-globin intron elements to consistently activate transgene expression in mice. These important intronic elements fail to transmit through lentivirus vectors designed for gene therapy of Sickle Cell Anemia. In this study, we identify intron modifications that reveal functional cooperation between the β-globin intronic enhancer and an intronic Oct-1 site. LCR activity in transgenic mice is also potentiated by an intronically located Igμ 3′MAR element. During induction of erythroid gene expression, the modified intron directs relocalization of the transgene away from the nuclear periphery towards more central neighbourhoods, and this movement mimics relocalization by the endogenous β-globin locus. Lentivirus vectors with the modified intron produce high titer virus stocks that express the transgene to therapeutic levels in erythroid cells. These findings have implications for understanding the mechanism of LCR activity, and for designing safe and effective lentivirus vectors for gene therapy.

## Introduction

The β-globin gene is regulated by a Locus Control Region (LCR) that interacts with gene proximal elements to activate erythroid specific expression. Many studies demonstrate that individual LCR hypersensitive sites (HS) loop out the intervening DNA to interact with the globin genes [1]–[4], and that transcriptional activation is accompanied by movement of the gene away from heterochromatin at the nuclear periphery towards transcription factories located more centrally [5]–[8]. While the LCR is not required to establish open chromatin at the endogenous locus [9], it is able to open chromatin at ectopic β-globin transgene integration sites to activate high level transcription [10]–[12]. This ability of the LCR, in particular its 5′HS3 element [13], to open chromatin at all integration sites has made it a widely used component of gene therapy expression cassettes designed for treatment of β-thalassemia or Sickle Cell Anemia. Several groups have corrected these diseases in mouse models using lentivirus mediated delivery of LCR β-globin vectors regulated, at least in part, by 5′HS3 [14]–[21].

One current limitation to LCR β-globin gene therapy cassettes is that high titer virus transmission requires the deletion of AT-rich (ATR) sequences in β-globin intron 2 [22],[23]. We have shown that these intronic ATR sequences are required for LCR activity by 5′HS3 when assayed in transgenic mice [24]. These data indicate that LCR β-globin sequences used in gene therapy cassettes may compromise chromatin opening and transcriptional enhancement activities. Thus, vectors containing the ATR deletion may require more than 1 integration per cell to ensure expression, as has been observed during gene therapy [25],[26]. Although increased vector copy number will direct therapeutic levels of expression, it also increases the likelihood of insertional activation of surrounding oncogenes [27]. In order to reduce potential genotoxicity, it would be advantageous to include insulator elements in the LTRs to block activation events [28]–[32] while also reducing the copy number load by redesigning the expression cassette to function more effectively [33].

To design improved expression cassettes, we dissected the ATR to discover functional elements that are not included in gene therapy cassettes [24]. The ATR contains binding sites for the erythroid specific factor Gata-1 and the ubiquitous factor Oct-1 [34],[35]. Mutation of these sites showed that Oct-1 is required for high level expression by 5′HS3 β/γ-globin hybrid transgenes, while the Gata-1 mutation had no effect [34]. The significance of Oct-1 on β-globin expression was further demonstrated in Oct-1 deficient mice generated by gene targeting [36], suggesting that an Oct-1 site should be incorporated into β-globin gene therapy cassettes.

The sequence within the ATR responsible for functionally interacting with 5′HS3 to open chromatin is unknown. A candidate element for this role is the Matrix Attachment Region (MAR) that overlaps the ATR [37],[38] but is deleted from gene therapy cassettes [22],[23]. This MAR contains 2 SATB1 (Special AT-Rich Binding-1) binding sites [35], and SATB1 is required for ε-globin expression in primitive erythroid cells [39]. We noticed that the 3′MAR in the immunoglobulin μ (Igμ) locus is also located in an intron and contains 3 SATB1 sites [40]. Importantly, the Igμ 3′MAR is able to transmit through a lentivirus and provides position independent expression in primary B lymphocytes [41]. These findings suggest that the Igμ 3′MAR might perform the same function when substituted for the β-globin ATR, and that this function could then be transmitted through a lentivirus vector.

Here, we introduce an Oct-1 consensus site and the Igμ 3′MAR into 5′HS3 β/γ-globin hybrid transgenes in which the ATR has been deleted. We show that the Oct-1 site cooperates with the β-globin intronic enhancer to increase expression levels in transgenic mice. The Igμ 3′MAR rescues LCR activity by 5′HS3 in single copy transgenic mice, and the combination of both an intronic Oct-1 site and Igμ 3′MAR in the BGT158 construct gives high expression levels nearly equivalent to those directed in the presence of the complete ATR. At a specific integration site in erythroid cells, the BGT158 construct is most responsive to transcriptional induction and spatially reorganizes into the nuclear interior. The endogenous β-globin locus also moves internally while ultrastructural heterochromatin organization remains primarily peripheral. Insulated lentivirus can be generated for all the 5′HS3 constructs with BGT158 giving the highest protein and RNA expression in erythroid cells. In addition, the modified BGT158 intron 2 increases the titer of vectors containing a larger LCR cassette (5′HS4, 5′HS3 and 5′HS2), although it is not essential for high level expression in this context. Overall, our data demonstrate the importance of an intronic Oct-1 site and MAR element to potentiate LCR activity by 5′HS3, show that a 5′HS3 β/γ-globin transgene undergoes normal nuclear relocalization during erythroid cell maturation, and provide evidence that modified introns are compatible with efficient lentivirus transduction resulting in robust Aγ-globin expression.

## Results

### Novel LCR β/γ-Globin Hybrid Transgenes with Intronic Oct-1 and MAR Elements

**Figure 1:**
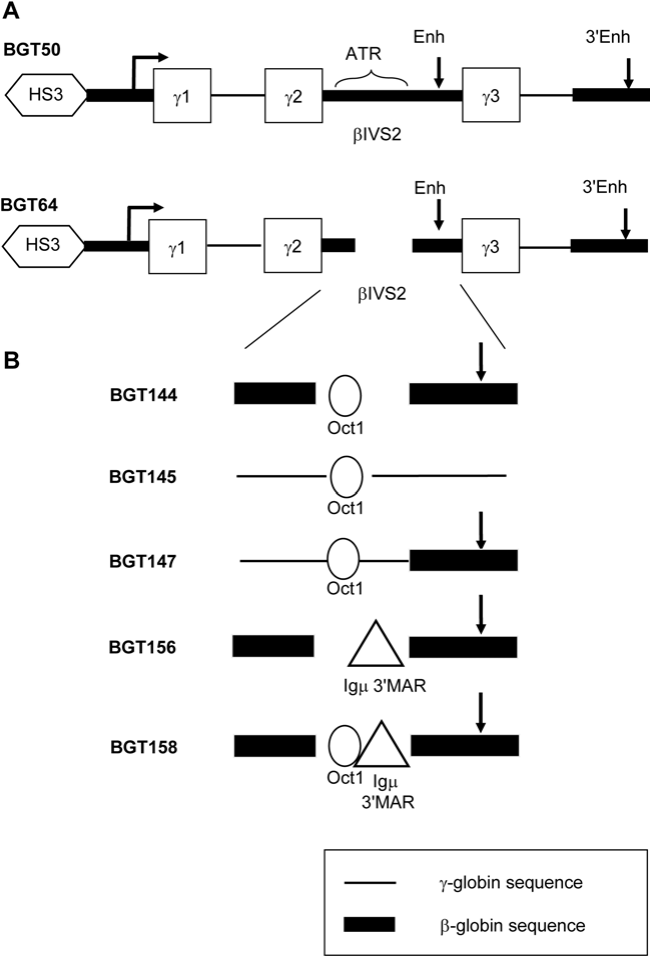
Maps of LCR β/γ-globin transgene constructs with intronic insertions of a consensus Oct-1 site or the Igμ 3′MAR element. (A) The BGT50 cassette is comprised of 5′HS3, β-globin promoter, Aγ-globin exons 1-3, β-globin intron 2 (βIVS2) containing the AT-rich region (ATR) and intronic enhancer, and the 3′ β-globin enhancer. The BGT64 cassette contains the 372 bp ATR deletion. (B) Intronic modifications generated in the LCR β/γ-globin transgenes. BGT144 contains an Oct-1 site in place of the deleted ATR along with the intronic enhancer. BGT145 has an Oct-1 site in the Aγ-globin intron 2. BGT147 contains the Oct-1 site and intronic enhancer in a hybrid intron with 5′ Aγ-globin and 3′ β-globin sequences. BGT156 contains the Igμ 3′MAR in place of the deleted ATR along with the intronic enhancer. BGT158 contains the Oct-1 site 5′ of the Igμ 3′MAR in place of the deleted ATR and the intronic enhancer.

We previously showed that the BGT64 transgene (Figure 1A) with a deletion of the 372 bp ATR directed reduced mean per copy expression of 31% and did not express at all integration sites in transgenic mice [24]. In contrast, the BGT50 transgene (Figure 1A) that contains the ATR expressed at all integration sites to a mean level of 64% per copy [24]. Since specific mutation of the Oct-1 site alone in the BGT131 transgene reduced mean expression to 31% but allowed expression at all sites [34], we reasoned that the Oct-1 site is important for high level expression and that some other component of the ATR functionally interacts with 5′HS3 LCR activity to direct expression at all integration sites. To create transgenes that more consistently express to high levels for use in virus vectors, we sought to reinsert a consensus Oct-1 site and a MAR element into the BGT64 transgene. In brief, BGT64 is composed of an 850 bp 5′HS3 element, the 815 bp β-globin promoter linked to Aγ-globin coding sequence and a downstream 260 bp β-globin 3′ enhancer. It also contains a β-globin intron 2 with the 372 bp ATR deletion (Figure 1). BGT64 encodes an Aγ-globin protein with 3 amino acids corresponding to β-globin due to coding sequences that flank β-globin intron 2.

To create improved transgenes, we first used site directed mutagenesis in order to encode completely wild-type Aγ-globin protein. We then introduced a consensus Oct-1 site and/or the Igμ 3′MAR element back into the site where the ATR was deleted. To determine if the β-globin intronic enhancer is functionally important, we inserted the Oct-1 site into the Aγ-globin intron 2 that lacks an enhancer in the BGT54 transgene (which also encodes completely wild-type Aγ-globin protein). Overall, 3 constructs were generated (Figure 1) to test if the Oct-1 site increases expression (BGT144) and cooperates with the intronic enhancer (BGT145,147). In addition, BGT156 was constructed to test the ability of the Igμ 3′MAR to rescue LCR activity by 5′HS3 at single copy integration sites, and BGT158 was used to examine whether the presence of both the Oct-1 site and Igμ 3′MAR results in more consistently high transgene expression.

To test the effect of these intronic modifications on expression by 5′HS3 β/γ-globin hybrid transgenes, they were microinjected into fertilized FVB mouse eggs. Fetuses were collected at day 15.5 and transgenic animals identified by DNA slot blot analysis. Southern blot analysis on fetal liver DNA determined transgene copy number, intactness and mosaicism (data not shown). Animals containing copy numbers >35, or mosaic animals containing <25% transgenic cells in the fetal liver, were excluded. Multicopy animals are sufficient for the purpose of defining mean per copy expression levels directed by the Oct-1 site since it is not required for single copy transgene expression, but LCR activity rescued by the Igμ 3′MAR requires analysis of at least 3 independent single copy mice.

### Oct-1 Site Rescues Expression Levels

**Figure 2:**
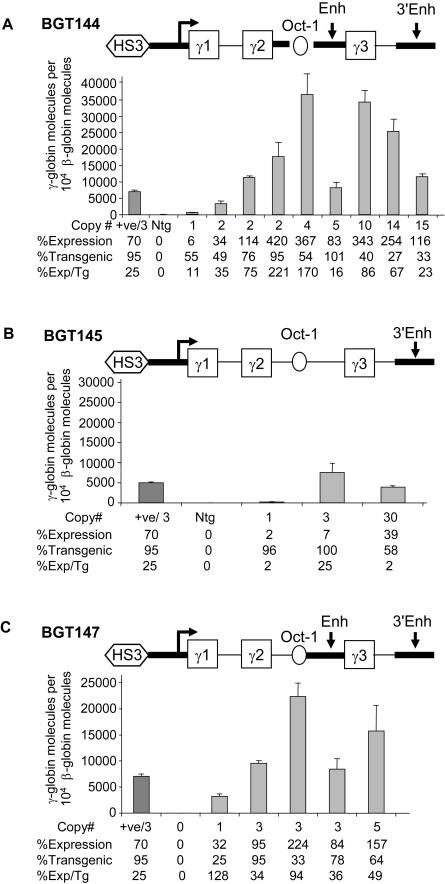
The Oct-1 site increases LCR β/γ-globin transgene expression by cooperating with the intronic enhancer. (A) RNA expression analysis using qRT-PCR on fetal liver tissue from BGT144 transgenic animals. The mean expression per transgene from qRT-PCR performed in triplicate for BGT144 is 78% ±24 (standard error-SE). (B) qRT-PCR results from BGT145 transgenic animals show that mean expression per transgene is 10% ±8. C) qRT-PCR results from BGT147 transgenic animals show that mean expression per transgene 68% ±18. In each case, the amount of product from amplification reactions with a primer set specific for the human β/γ-globin transcript was scaled relative to 10^4^ molecules from amplification reaction with primers specific for the mouse βmajor-globin gene. The positive control (+ve) is a previously published BGT64 sample (FF334) and the negative control (Ntg) is a nontransgenic animal.

A total of 9 transgenic animals were identified with intact BGT144 transgenes, ranging in copy number from 1 to 15. qRT-PCR was performed on fetal liver RNA to determine expression levels of the transgene relative to endogenous mouse βmajor-globin (Figure 2A). As controls for the accuracy of the RT-PCR reactions, a previously analyzed BGT64 sample (+ve) that expressed at 23% per mouse βmajor-globin gene by S1 nuclease assay [24] and a non-transgenic (Ntg) sample were analyzed. BGT144 animals express the transgene to a mean per copy level of 78% ±24 (SE). These results are significantly different from previous BGT64 results (P = 0.05 by two-sided Mann-Whitney test) and demonstrate that the Oct-1 site rescues mean expression levels from transgenes that lack the ATR.

### Oct-1 Site Cooperates with the Intronic Enhancer

To evaluate whether the Oct-1 site cooperates with the intronic enhancer to rescue expression levels, transgenic animals were generated with the BGT145 and BGT147 constructs. Three transgenic animals were identified with intact BGT145 transgenes ranging in copy number from 1 to 30. qRT-PCR analysis showed that BGT145 animals express the transgene to a mean per copy level of 10% ±8 (Figure 2B). These data suggest that Oct-1 does not rescue expression levels when inserted into an Aγ-globin intron 2 that lacks an intronic enhancer. The BGT147 transgene contains a hybrid intron that includes the β-globin intronic enhancer (Figure 2C). Five transgenic animals were identified with intact BGT147 transgenes ranging in copy number from 1 to 5. qRT-PCR demonstrated that BGT147 animals express the transgene to a mean per copy level of 68% ±18 with a significance of P = 0.01 (Figure 2C). These data confirm that the Oct-1 site cooperates with the intronic enhancer to increase expression levels, even within the context of a hybrid intron 2.

### Igμ 3′MAR Rescues LCR Activity but with Variable Expression

**Figure 3:**
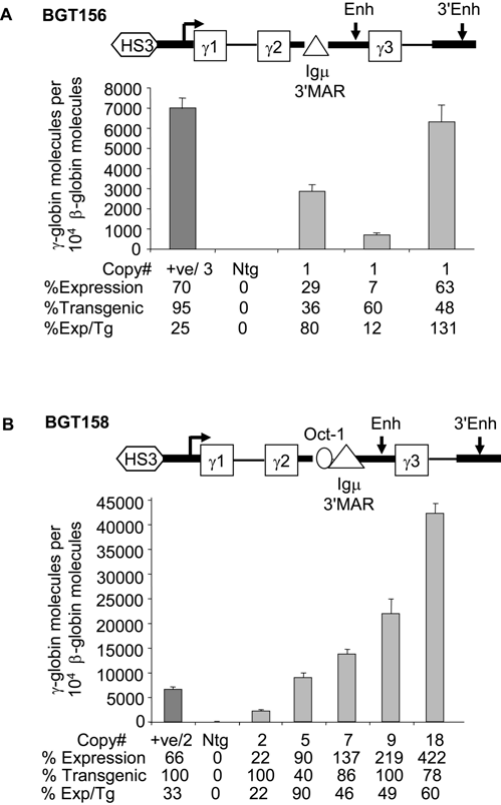
The Igμ 3′MAR rescues LCR activity but consistent expression levels requires the Oct-1 site. (A) qRT-PCR results from BGT156 transgenic animals show mean expression per transgene is 74% ±34. (B) qRT-PCR results from BGT158 transgenic animals show mean expression per transgene is 53% ±11. All samples analyzed as described in Figure 2, with the exception that the positive control (+ve) in BGT158 is fetal liver RNA from a previously characterized bred homozygous BGT50 mouse line.

To test if replacement of the β-globin ATR with the Igμ 3′MAR rescues LCR activity in single copy transgenic mice, the BGT156 cassette was generated. Three single copy transgenic animals were identified with intact BGT156 transgenes, but no multicopy animals were obtained. The BGT156 single copy animals express the transgene to a mean per copy level of 74% ±34 (Figure 3A) but with quite variable expression output (range 12–131%). These results indicate that the Igμ 3′MAR rescues 5′HS3 directed LCR activity at single copy.

### Oct-1 Site and Igμ 3′MAR Direct Consistent Expression

To test if introduction of the Oct-1 site upstream of the Igμ 3′MAR helps direct more consistently high Aγ-globin expression, the BGT158 construct was evaluated. Five transgenic animals were identified with intact BGT158 transgenes ranging in copy number from 2 to 18. As controls for the accuracy of the PCR reactions, a previously analysed BGT50 homozygous fetal liver sample (+ve) that expressed at 40% per mouse βmajor-globin gene by S1 nuclease assay was used [24]. BGT158 animals express the transgene to a mean level of 53% ±11 with a significance of P = 0.01 (Figure 3B). These findings suggest that the BGT158 transgene directs high levels of consistent expression regardless of the integration site. Activities associated with the Oct-1 site, Igμ 3′MAR and intronic enhancer contribute to this effect. Because no single copy animals were identified with the BGT158 construct to directly compare its expression with the single copy BGT156 animals, we proceeded to insert single copies of both these constructs into the same genomic site in Mouse Erythroleukemia (MEL) cells.

### Integration and Induction of LCR β/γ-Globin Transgenes at the Same Genomic Site

**Figure 4:**
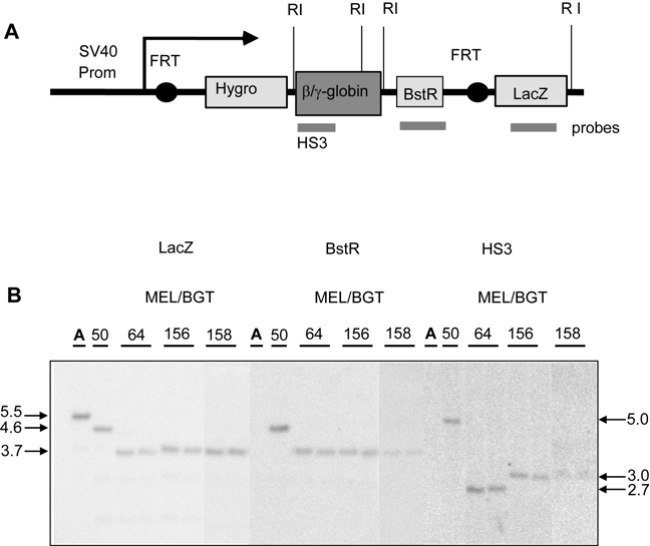
Single copy integration of LCR β/γ-globin transgenes into a specific FRT site in MEL acceptor cells. (A) Structure of the transgene after targeted insertion in MEL cells showing the location of *Eco*RI (R1) restriction sites and the LacZ, BstR and HS3 probes (grey lines). MEL acceptor DNA is cut 3′ of LacZ, and BGT50 is cut 3′ of LacZ and once within the β/γ-globin sequence. Two additional *Eco*RI sites were introduced in the 5′ and 3′ linkers during SFV vector subcloning of BGT64, BGT156, BGT158. (B) Southern blot analysis of genomic DNA cut with *Eco*RI. Hybridization with LacZ, BstR and HS3 probes confirms intactness of the integrated constructs. Hybridization with BstR and HS3 probes detects no band in the MEL acceptor cell line as expected, or a single band of the expected size for a single site transgene integration at the FRT site and the absence of random integrations.

We took advantage of the FLP recombinase system to direct insertion of BGT156, BGT158 and BGT64 as a control into the same FRT site in MEL cells (Figure 4A). The BGT50 transgene had previously been inserted into this same site [42]. Proper integration at the FRT site was verified for 2 independent BGT156, BGT158 and BGT64 MEL cell clones by Southern blot analysis using a LacZ probe (Figure 4B). Digestion with *Eco*RI which cuts MEL acceptor genomic DNA downstream of LacZ detected a single band of 5.5 kb in the MEL acceptor cell line. The BGT50 cell line has an additional *Eco*RI site in the 5′HS3 β/γ-globin hybrid sequence and produced the expected single 4.6 kb band. Two additional flanking *Eco*RI sites were introduced during subcloning of BGT156, BGT158 and BGT64 into the SFV plasmid resulting in detection of a common 3.7 kb band by the LacZ probe. Intactness of 5′HS3 β/γ-globin sequences and single site insertion was verified with the BstR probe and the internal HS3 probe that reveal the expected band sizes, with the 5′HS3 fragment of BGT64 being smaller because of its intron 2 deletion (Figure 4B). Together these data demonstrate that integration of the transgenes occurred at the FRT site with no randomly integrated vector DNA.

**Figure 5:**
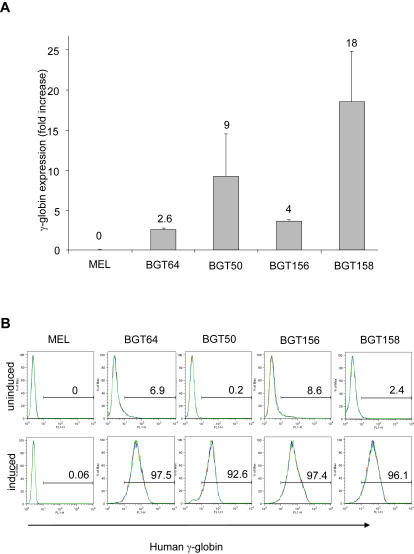
The BGT158 transgene responds with the highest fold induction in MEL cells. (A) qRT-PCR analysis of Aγ-globin RNA expression by MEL BGT64, MEL BGT156 and MEL BGT158 cell lines. Mean fold increase of RNA levels ±SE is shown on the third day after induction with 5 mM HMBA calculated from triplicate qRT-PCR samples from 2 independent clones for each transgene. MEL and MEL BGT50 cell lines are negative and positive controls respectively. (B) Flow cytometry with and without 6 days of HMBA induction shows the cell % expressing Aγ-globin protein, and demonstrates that all constructs produce roughly equivalent protein levels.

To assess expression of Aγ-globin in these cell lines, we measured RNA levels before and after induction with 5 mM HMBA. RNA was obtained from the 2 independent cell clones for each construct and subjected to qRT-PCR in triplicate. Expression of Aγ-globin transcripts was normalized to PolII large subunit transcripts. After induction for 3 days, the mean amount of BGT64 RNA increased just 2.6 fold while BGT50 and BGT158 RNA increased 9 fold and 18 fold respectively (Figure 5A). In contrast, BGT156 transcripts increased just 4 fold, due to relatively high levels of transcripts in the uninduced cells. These data indicate that BGT156 expresses prematurely at this integration site. In contrast, the greater fold RNA induction of BGT158 reflects its low expression in uninduced cells. To ensure equivalent induction quality for the different cell lines, flow cytometry was performed at day 6 to allow accumulation of Aγ-globin protein (Figure 5B). This analysis demonstrates that uninduced BGT50 and BGT158 cells do not express Aγ-globin protein, but 7–8% of BGT64 and BGT156 cells are Aγ-globin positive which is consistent with the RNA analysis. After induction, the BGT156 and BGT158 constructs express Aγ-globin protein to similar levels. These results demonstrate that the BGT158 construct is more finely regulated than BGT64 and BGT156 at this integration site.

### Nuclear Relocalization of the BGT158 Transgene during MEL Cell Induction

**Figure 6:**
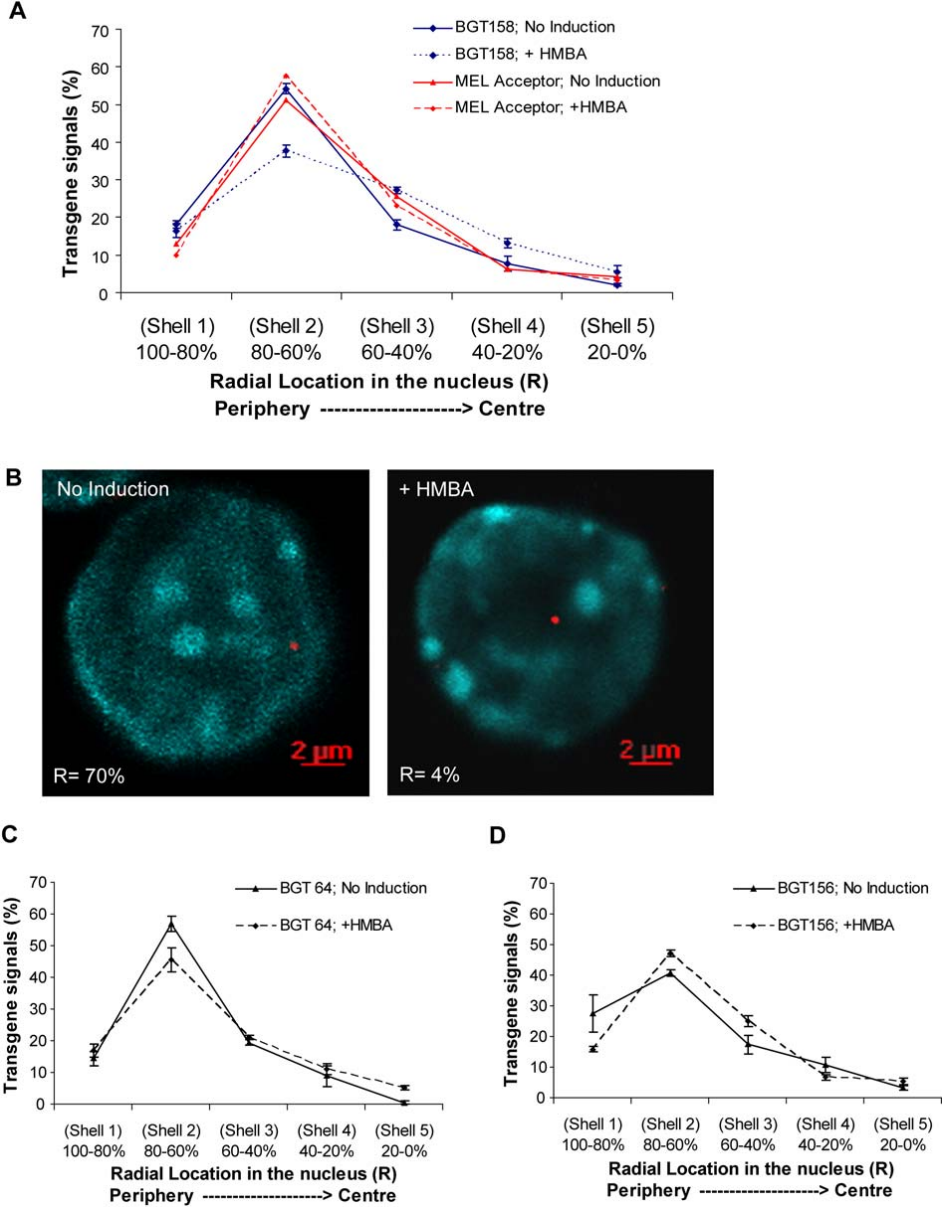
Nuclear relocalization of the BGT158 transgene in MEL cells during HMBA induction. (A) Radial distribution of BGT158 transgene examined by 3D DNA FISH. The x-axis indicates percentage of the nuclear radius, in which 100-80% of the nucleus represents the periphery and 20-0% represents the centre. Each condition was repeated in three individual experiments with at least 80 transgene signals scored for each experiment. The data shown is the mean from all 3 experiments ±SE. (B) Examples of BGT158 transgene localization by 3D DNA FISH, with or without HMBA induction. The z-section containing the transgene signal used for radial measurements is shown. The transgene is detected in red with DIG labeled probe and tyramide signal amplication, and nuclear DNA is counterstained with DAPI (cyan). The number at the bottom of each panel represents the radial location in the nucleus shown. (C) Radial distribution of BGT64 and BGT156 transgenes examined by 3D DNA FISH as described in A above. BGT156 was repeated in three individual experiments with greater than 250 transgene signals scored. BGT64 was repeated in two individual experiments with greater than 160 transgene signals scored for each experiment.

To determine whether 5′HS3 β/γ-globin transgenes are properly localized within the nucleus, we performed three-dimensional DNA FISH (fluorescent in-situ hybridization) with and without 3 days of HMBA induction. We chose the MEL BGT158 cells for this analysis because they have the greatest induction of RNA over this period. To detect the BGT158 transgene by FISH, the 10 kb SFV plasmid used for FRT site-specific insertion was labeled with digoxigenin (DIG) and the probe signal was amplified using Alexa Fluor 546 labelled tyramide [43]. FISH signals were detected on BGT158 transgenes (see 3D reconstruction in Video S1), but not in the MEL acceptor cell line containing the FRT site (data not shown). Measurements of the mean radial distribution of BGT158 transgenes in >80 nuclei from each of 3 experiments were binned into 5 radial shells extending from shell 1 at the periphery to shell 5 at the centre (Figure 6A and 6B). The z-sections used for these measurements had the same average nuclear diameter in both the uninduced and induced samples. In uninduced cells, the majority of BGT158 transgenes (54%) are detected in shell 2, near but not at the nuclear periphery. After HMBA induction, BGT158 transgene localization in shell 2 falls to 37% with a concomitant increase in its frequency in more internal shells. Due to the high cumulative n = 250 nuclei from 3 separate experiments, these results are significant at P<0.001 by two-sided Mann-Whitney test. At the same time we measured transgene proximity to the nearest DAPI-rich heterochromatin. Approximately 62% of BGT158 transgene signals are within 1 µm of DAPI-rich heterochromatin in the uninduced state, but HMBA induction only slightly reduces this proximity to 51% (Figure S1). To confirm that radial relocalization is dependent on BGT158 sequences and is not a consequence of the FRT integration site, we repeated the experiment using a 3.0 kb LacZ fragment as probe on the original MEL acceptor cell line. In this case there was no relocalization after induction with greater than 50% of the signal remaining in shell 2 (P = 0.92) (Figure 6A). These data document relocalization of the BGT158 transgene towards more internal nuclear positions during globin gene induction. To extend these results, relocalization of the globin transgene was tracked using the same 3D DNA FISH probe in BGT64 and BGT156 cells (Figure 6C and 6D). In the absence of the intron 2 ATR, the BGT64 transgene preferentially localized to shell 2. After induction there was a slight reduction in shell 2 occupation but without any obvious preference for moving internally (P = 0.35). BGT156 transgenes localized in shells 1 and 2 before moving internally into shells 2 and 3 upon induction (P = 0.04). We conclude that the intron 2 ATR contributes to nuclear relocalization during erythroid induction. The presence of the Igμ 3′MAR alone facilitates a less pronounced relocalization during induction than observed with the BGT158 construct.

### Nuclear Relocalization of the Endogenous β-Globin Locus during MEL Cell Induction

**Figure 7:**
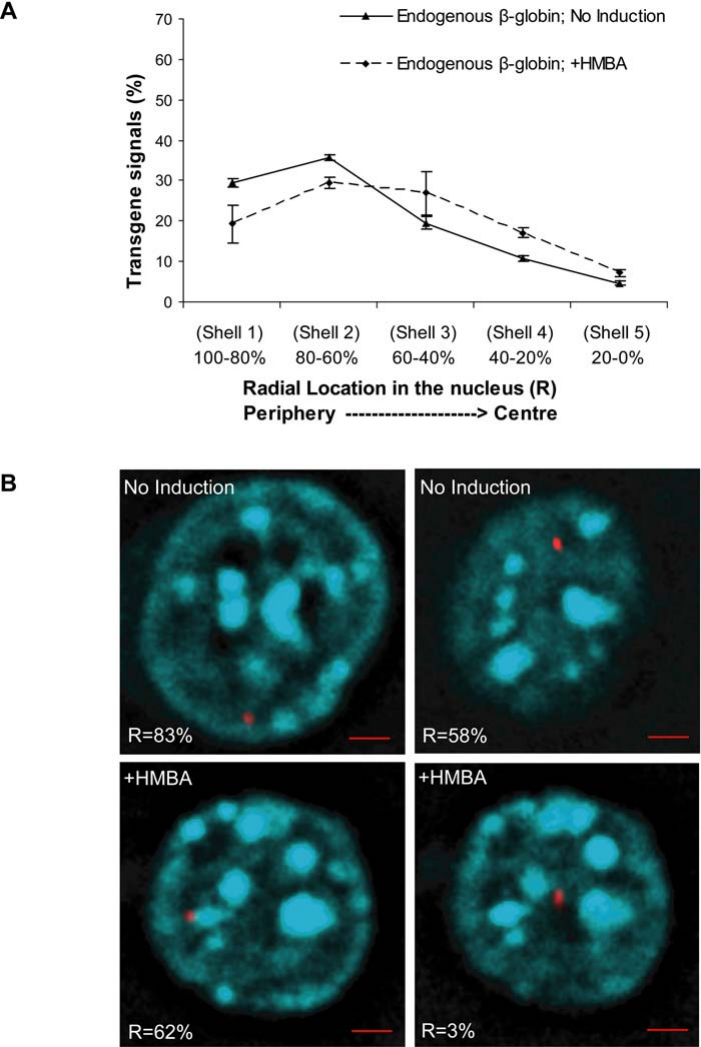
Nuclear relocalization of the endogenous β-globin locus in MEL cells during HMBA induction. (A) Radial distribution of the endogenous mouse β-globin locus examined by 3D DNA FISH as described in Figure 6. Each condition was repeated in three individual experiments with greater than 250 transgene signals scored. (B) Examples of endogenous mouse β-globin locus localization by 3D DNA FISH, with or without HMBA induction. Two z-sections from the same cell containing one FISH signal each are shown and were used for radial measurements. The transgene is detected in red with DIG labeled BAC probe, and nuclear DNA is counterstained with DAPI (cyan). The number at the bottom of each panel represents the radial location in the nucleus shown. Red scale bars indicate 2 µm.

To determine whether the endogenous mouse β-globin locus also relocalizes from the periphery to more central locations upon induction, we performed 3D DNA FISH with a BAC probe but without tyramide amplification (Figure 7A and 7B). This analysis detected a preference for shells 1 and 2 and movement into more interior shells after induction (P<0.001). This movement of the endogenous β-globin locus is similar to the movement of the BGT158 transgene, and when induced they both resemble the localization of the endogenous mouse β-globin locus in fetal liver cells at stage 3 (CD117-, Ery1+, Ter119+) of erythroid development [8]. Flow cytometry demonstrates that uninduced MEL cells are CD117-, Ery1^lo^, CD71+ and Ter119- (Figure S2). This may represent the stage 2/3 transition where CD117 has turned off, Ery1 is activated to some degree but Ter119 has not yet turned on. Upon induction, MEL cells become CD117-, Ery1-, CD71^lo^ but remain Ter119- and therefore cannot be directly compared with the known Ter119+ late stages of erythroid development.

### Ultrastructural Organization of Heterochromatin during MEL Cell Induction

**Figure 8:**
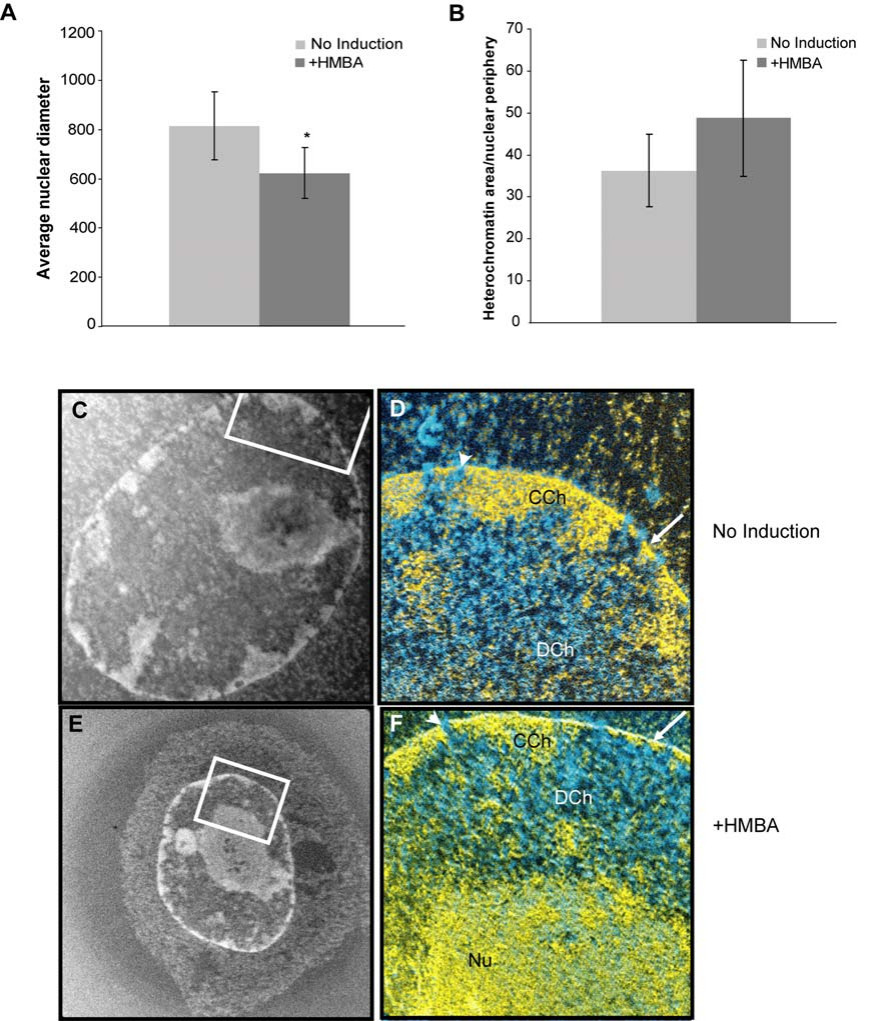
Nuclear organization of ultrastructural heterochromatin in MEL cells during HMBA induction. Electron Spectroscopic Imaging (ESI) analysis of undifferentiated and three-day differentiated MEL cells. (A) The average diameter of undifferentiated MEL cells changes upon HMBA differentiation. The diameters of sectioned cells were measured as described in the methods section. (B) The change in size is correlated to a small and statistically insignificant change in peripheral heterochromatin distribution after differentiation. Low magnification phosphorus enhanced mass images of a large undifferentiated nucleus (C) and a small differentiated (E) nucleus. Higher-resolution element specific ratio maps were acquired of regions of interest indicated by white boxes. Phosphorus element specific ratio maps were pseudo-coloured yellow and overlaid onto phosphorus subtracted nitrogen maps pseudo-coloured cyan. Phosphorus containing chromatin appears yellow and protein-rich regions are blue. An example of condensed chromatin regions are indicated (CCh) in both the undifferentiated (D) and differentiated (F) cells as is a decondensed region (DCh), the nucleolus is demarked by Nu and nuclear pores with a bold arrowhead and thinly represented condensed chromatin at the envelope with a white arrow.

To examine heterochromatin organization at the ultrastructural level in single MEL cells, we performed Electron Spectroscopic Imaging (ESI) [44]. This technique allows visualization of chromatin fibers after electron microscopy of 70 nm cell sections. We first examined the effect of 3 days HMBA induction on nuclear volume in low magnification phosphorus enhanced mass images (Figure 8A). While there is a range of diameters measured in the random sections chosen, the average nuclear diameter of induced cells is reduced by 24% compared to untreated controls and thus the average nuclear volume is significantly smaller. This size reduction could potentially result in a global compaction of chromatin. To address whether the volume change leads to an increase in the amount of condensed chromatin, we measured the area of condensed chromatin along the nuclear envelope (Figure 8B). These areas from each cell were normalized to the perimeter corresponding to the area measured to provide a measurement of compaction of chromatin along the nuclear envelope. We found that there was a slight increase in the average amount of peripheral heterochromatin, though this difference was not statistically significant. The vast majority of the condensed chromatin within these cells is located at the periphery, or adjacent to the nucleolus, with a moderate subset localizing to the regions surrounding the nucleolus. To represent the most extreme differences in nuclear diameter, we chose to compare one of the largest uninduced nuclei to one of the smallest induced nuclei (Figure 8C–8F). Qualitative analysis of the high magnification phosphorus (P) and nitrogen (N) images indicates that thickened regions of condensed chromatin (CCh) along the nuclear envelope are interspersed with regions where the condensed chromatin is thinly represented (arrow) and where nuclear pores can be easily visualized (arrowhead). The remainder of the chromatin throughout the nucleoplasmic volume is represented by relatively decondensed (DCh) and sparsely represented chromatin fibers. The volume not represented by chromatin (blue) has decreased by differentiation but the structural features of the decondensed and the condensed chromatin is unaffected. Thus, the nuclear envelope is lined with ultrastructural heterochromatin in both conditions and transgene movement detected by 3D DNA FISH is not the result of heterochromatin reorganization during MEL cell induction.

### Lentivirus Vector Construction and Expression in MEL Cells

**Figure 9:**
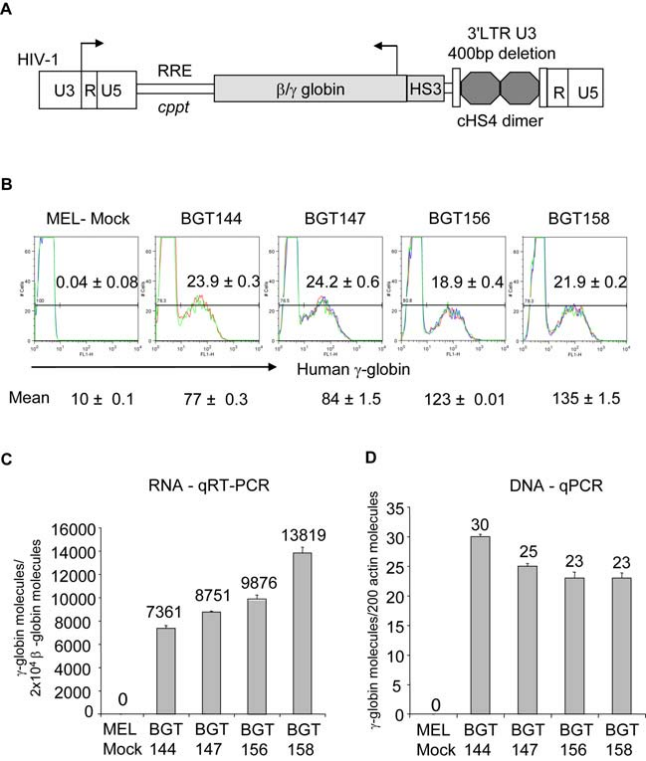
Insulated SIN lentivirus vectors containing 5′HS3 β/γ-globin transgenes express in MEL cells. (A) Map of the PL.SIN.cHS4 lentivirus showing the HIV 5′LTR, cppt, RRE, 5′HS3 β/γ-globin transgene and the 400 bp SIN deletion of the 3′LTR bearing the dimer core cHS4 Insulator. (B) Lentivirus vectors containing the intronic Igμ 3′MAR express the highest mean Aγ-globin protein levels assessed by flow cytometry of MEL cells infected at low MOI. Data with approximately 20% Aγ-globin protein expressing cells is shown by flow cytometry performed in triplicate ±SE after 6 days induction with HMBA. (C) Lentivirus vectors containing the intronic Igμ 3′MAR express the highest Aγ-globin RNA levels. qRT-PCR results in triplicate ±SE were performed on the 20% protein positive infected MEL cells after 3 days induction with HMBA. RNA expression is shown as Aγ-globin molecules per 2×10^4^ mouse βmajor-globin molecules after correction for the lentivirus copy number. (D) The lentivirus copy number is approximately equal in each infection. qPCR analysis of lentivirus copy number ±SE in the MEL cell genome. The number of transgene molecules was determined by amplification with 3′ β-globin enhancer primers and plotted versus 200 mouse β-actin molecules. These data correspond to the % of infected MEL cells.

Given that BGT158 is properly regulated with normal nuclear dynamics and high Aγ-globin protein accumulation in MEL acceptor cells, we proceeded to test whether it functioned better than the other constructs in gene therapy vectors. As all the constructs were designed to be transmitted through virus vectors, we inserted them in the antisense orientation into a SIN lentivirus vector containing a dimer core cHS4 insulator element in the 3′LTR (Figure 9A). The BGT145 construct was excluded from this analysis due its low level Aγ-globin RNA expression in transgenic mice (Figure 2B). These lentiviral vectors were concentrated 250 fold by ultracentrifugation. To analyze expression of Aγ-globin, MEL cells were infected with 4 to 40 µl of concentrated lentivirus and induced with 5 mM HMBA. DNA and RNA samples were collected on day 3, with flow cytometry performed in triplicate on day 6 to calculate titers of Aγ-globin protein expressing virus. Virus titers per ml from three independent inductions were as follows: BGT144 (4.8×10^6^), BGT147 (1.2×10^6^), BGT156 (3.8×10^6^) and BGT158 (1.1×10^7^). Samples with approximately 20% Aγ-globin expressing cells containing roughly equal vector copy numbers are shown (Figure 9B). This analysis reveals that BGT156 and BGT158 lentivirus express the highest levels of Aγ-globin protein at 123 and 135 mean fluorescence units (MFI) respectively, while BGT144 and BGT147 express only 77 and 84 MFI.

To determine the amounts of Aγ-globin transcripts in the infected MEL cells, qRT-PCR was performed in triplicate relative to 2×10^4^ mouse βmajor-globin molecules, after correction for lentivirus copy number deduced by qPCR relative to the mouse β-actin gene. These results parallel the protein analysis, with higher Aγ-globin transcript levels detected for BGT156 and BGT158 at 0.99×10^4^ and 1.38×10^4^ molecules, in comparison to 0.74×10^4^ and 0.88×10^4^ molecules for BGT144 and BGT147 (Figure 9C). Lentivirus copy numbers demonstrate that all vectors infected between 23–30% of the cells (Figure 9D). As reported by others, PCR amplification of the dimer core cHS4 in the LTRs shows that it is reduced to a monomer after transduction (Figure S3). We conclude that BGT158 is the most efficient vector because it expresses the highest Aγ-globin RNA and protein levels from the lowest number of infected cells. Overall BGT158 lentivirus transcripts are 65% the level of mouse βmajor-globin in MEL cells.

### Increased Titer by the BGT158 Intron and Complementation by Other HS Elements

**Figure 10:**
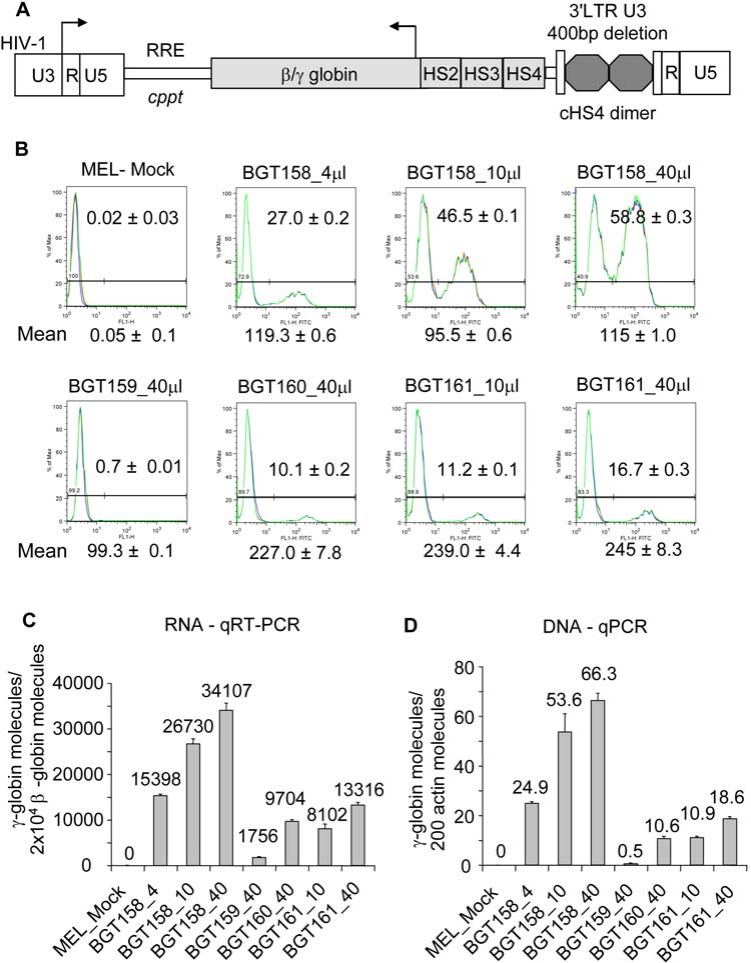
Increased titer by the BGT158 intron 2 and complementation by other HS elements. (A) Map of the PL.SIN.cHS4 lentivirus showing the LCR (5′HS2-4) β/γ-globin transgene and other components described in Figure 9. BGT159 contains the wild-type intron 2 from BGT50, BGT160 contains the BGT64 intron 2 with the deleted ATR (Figure 1A), and BGT161 contains the BGT158 modified intron 2 (Figure 1B). (B) The presence of 5′HS4 and 5′HS2 compensates for the absence of the intron 2 ATR in BGT160. BGT160 expresses high mean Aγ-globin protein levels assessed by flow cytometry of MEL cells performed in triplicate ±SE after 6 days induction with HMBA. The presence of the BGT158 modified intron 2 in BGT161 increases the viral titer but not the MFI. (C) qRT-PCR results in triplicate ±SE were performed on the infected MEL cells after 3 days induction with HMBA. RNA expression is shown as Aγ-globin molecules per 2×10^4^ mouse βmajor-globin molecules after correction for the lentivirus copy number. (D) The lentivirus copy number (detected by qPCR analysis as described in Figure 9D) is approximately equal to the % of Aγ-globin+ cells detected by flow cytometry.

It has recently been reported that the 5′HS4 element of the LCR contributes to high expression levels in β-globin lentivirus vectors and that the functional part of this element can be substituted by the IFNβ S/MAR in the absence of the intron 2 ATR [45]. To determine how the modified BGT158 intron 2 might influence expression of transgenes that also include 5′HS4 and 5′HS2, we generated insulated lentivirus vectors with the 3.0 kb BGT14 LCR [46] composed of 5′HS4, 5′HS3 and 5′HS2 (Figure 10A). These vectors have different intron 2 compositions; BGT159 contains the wild-type intron 2 from BGT50, BGT160 contains the intron 2 ATR deletion from BGT64, and BGT161 contains the modified BGT158 intron 2. Infections of MEL cells with 4 to 40 µl of virus were performed for comparison with the BGT158 lentivirus. Flow cytometry for Aγ-globin expression (Figure 10B) demonstrates that BGT159 fails to make significant virus titers per ml (<6×10^4^) as expected for the wild-type intron 2. In contrast, BGT160 and BGT161 produce high and roughly equivalent levels of Aγ-globin protein (MFI 270 and 239) that exceed the levels from BGT158 (MFI 119), suggesting that 5′HS4 or 5′HS2 can compensate for the absence of the intron 2 ATR. Nevertheless, the titer of the BGT158 virus is much greater (1.4×10^7^) than BGT160 (5.2×10^5^). The presence of the modified intron 2 in BGT161 increased the titer to 2.3×10^6^. With respect to RNA levels (Figure 10C), the sample infected with 4 µl of BGT158 virus produced 1.54×10^4^ Aγ-globin molecules (75% of mouse β-globin), while 40 µl of BGT160 produced 0.97×10^4^ (48%) and 10 µl of BGT161 produced 0.81×10^4^ molecules (40%). DNA analysis reveals a precise correspondence between provirus content (Figure 10D) and the percentage of expressing cells (Figure 10B). We conclude that the larger LCR cassette can compensate for the absence of the intron 2 ATR in MEL cells, but that the modified BGT158 intron 2 increases the titer of viruses with the larger LCR. In summary, the use of this modified intron 2 in 5′HS3 and larger LCR constructs including 5′HS4 and 5′HS2 should facilitate consistent expression at single vector copy during gene therapy of hemoglobinopathies.

## Discussion

LCR β-globin transgenes used for gene therapy of hemoglobinopathies contain a deletion in intron 2 that removes functionally important ATR sequences and may compromise expression. Here we regenerate the intron and demonstrate the ability of these transgenes to transmit through an insulated SIN lentivirus vector. Using different combinations of intronic elements, we show that the Oct-1 site cooperates with the β-globin intronic enhancer to raise the mean level of expression in transgenic mice, and that the Igμ 3′MAR can functionally interact with 5′HS3 to direct LCR activity at single copy integration sites. The BGT158 construct containing all of these elements shows good induction in erythroid cells with normal nuclear relocalization dynamics. Moreover, BGT158 transmits at high titer through lentivirus vectors and expresses robust Aγ-globin levels in erythroid cells. These findings have implications for understanding the mechanism of LCR activity at ectopic transgene integration sites, and for designing lentivirus vectors with minimal genotoxicity risk for gene therapy.

### Oct-1 Site Cooperates with the Intronic Enhancer To Rescue Expression Levels

To create transgenes that express highly and transmit through viral vectors, we inserted a consensus Oct-1 site into intron 2 of 5′HS3 β/γ-globin constructs that lack the ATR. The BGT144 and BGT147 constructs expressed Aγ-globin transcripts to a mean per copy level of 78% ±24 and 68% ±18 respectively. These results demonstrate the functional importance of the Oct-1 site in rescuing expression levels in the presence of the intronic enhancer. This conclusion is consistent with our previous observation that mutation of the Oct-1 site alone in the BGT131 transgene reduces expression levels [34]. However, the BGT145 construct contains the Oct-1 site in an Aγ-globin intron 2 that lacks an enhancer, and mean per copy transgene expression was reduced to 10% ±8. These findings strongly indicate that the Oct-1 site alone is not sufficient to activate high expression. We conclude that the Oct-1 site must cooperate with the intronic enhancer. This enhancer is composed of three Gata-1 sites that form a DNaseI hypersensitive site in erythroid cells [47]. Since both Oct-1 and Gata-1 can act as either transcriptional activators or repressors depending on the context [48]–[50], one interpretation of our results is that the transgene has reduced activation or may even be actively repressed when only one of the factors is bound to the intron.

### Igμ 3′MAR Potentiates 5′HS3 LCR Activity at Single Copy

To test the functional importance of a MAR in LCR activity, we generated a construct in which the ATR is replaced by the Igμ 3′MAR. Expression from 3 of 3 single copy BGT156 mice at a mean per copy level of 74% ±34 demonstrates that the Igμ 3′MAR functionally interacts with 5′HS3 to direct LCR activity at ectopic integration sites. This ability to open chromatin does not require the Oct-1 site, and is consistent with expression from 3 of 3 single copy BGT131 transgenic mice and 7 of 7 single copy BGT133 animals in which the Oct-1 site is mutated but the β-globin intron 2 MAR is intact [34]. However, BGT131 and BGT133 mean per copy expression falls to 31–37%, indicating that the Igμ 3′MAR is a stronger positive element than the β-globin intron 2 MAR. The Igμ 3′MAR has 3 SATB1 binding sites and promotes extension of open chromatin from the adjacent Igμ enhancer [51]. SATB1 acts as a landing platform for chromatin remodeling factors [43],[52], mediates DNA looping [53],[54], and has been implicated in ε-globin gene regulation [39]. Since the Igμ 3′MAR and the β-globin intron 2 MAR have similar locations near intronic enhancers and are bound by SATB1, it is likely that they perform similar functions in extending open chromatin to potentiate enhancer or LCR function. Both of these MARs lie in regions that influence initiation of DNA replication and its timing may be associated with the chromatin state [55],[56], or they could participate in DNA looping events that accompany gene activation [57]. For example, 3C results show that in the Activated Chromatin Hub 5′HS3 loops to interact with a region in the β-globin gene that is not the promoter [4], and MARs or HS that flank the β-globin locus may also contribute to Chromatin Hub formation in progenitor cells [38],[58]. It is important to acknowledge that other factors bind to the Igμ 3′MAR and may contribute to its stronger activity in the context of 5′HS3 β/γ-globin transgenes [59].

In our final BGT158 construct, we added the Oct-1 site to the Igμ 3′MAR and obtained mean per copy expression of 53% ±11. Thus, BGT158 expression is more consistent (range 22–90%) but its mean per copy level is less than BGT156. These results suggest that the combination of the Oct-1 site and intronic enhancer moderates the activity of Igμ 3′MAR at ectopic sites in erythroid cells. At the immunoglobulin locus, the Igμ 3′MAR is also adjacent to an intronic enhancer that contains an Oct-1 site [40].

### BGT158 Transgene Relocalizes to the Nuclear Interior during Transcriptional Induction

To compare BGT156 and BGT158 transgene activity at the same single copy integration site, we inserted them into a specific FRT site in erythroid cells. Analysis of transcriptional induction demonstrated that the BGT156 transgene is prematurely expressed and therefore induces only 4 fold, while the BGT158 construct has an 18 fold response. These data indicate that the Oct-1 site in BGT158 also moderates the Igμ 3′MAR in uninduced erythroid cells leading to more precise regulation. β-globin induction in MEL cells correlates with increasing levels of the NF-E2 erythroid specific factor [60] which binds just outside the 5′HS3 core but in the 5′HS2 core [61],[62]. NF-E2 is implicated in relocation of 5′HS2 regulated β-globin genes to the nuclear interior upon MEL cell induction [60] and may repress the LCR in uninduced cells [63]. Induction of BGT158 shows that 5′HS3 regulated transgenes also perform normal nuclear relocalization that resembles movement of the endogenous β-globin locus. However, BGT158 movement away from DAPI-rich heterochromatin is not as pronounced as that reported with 5′HS2 regulated genes. This difference may reflect that 5′HS3 does not have the strong enhancer activity present in 5′HS2 that is responsible for movement away from heterochromatin [7]. This NF-E2 enhancement activity is not required to form the ACH [64]. In contrast, Gata-1 and EKLF factors that bind the 5′HS3 core do participate in ACH formation [65],[66]. Current models of LCR function suggest that movement to the nuclear interior by the 5′HS3 transgenes may reflect preferred engagement domains with transcription factories [6],[8]. Our ESI experiments demonstrate that the nuclear interior is rich in ultrastructural euchromatin and that heterochromatin located at the periphery does not reorganize during MEL cell differentiation.

### Transgene and Lentivirus Vector Design for Gene Therapy

The BGT158 transgene is a novel construct designed to investigate LCR activity and for use in gene therapy. It is optimized to express wild-type Aγ-globin protein while incorporating functional intronic elements that cooperate to rescue expression levels and LCR activity from 5′HS3. To minimize potential genotoxicity, we inserted BGT158 into a cHS4 insulated SIN lentivirus vector. Infections performed at low MOI demonstrate that it expresses highly in erythroid cells, and confirm that the Igμ 3′MAR is able to transmit efficiently through lentivirus vectors [41]. In the context of the larger LCR element that includes 5′HS4, 5′HS3 and 5′HS2, the modified BGT158 intron 2 is not essential for high level protein expression in MEL cells. As 5′HS4 may contain a MAR element, this activity may functionally compensate for the intron 2 MAR [45]. Nevertheless, the modified intron 2 raised the titer of both the 5′HS3 and larger LCR cassette vectors. Our prior single copy transgenic mouse data indicates that 5′HS3 regulated transgenes [24] express to higher levels in vivo than LCR cassettes containing 5′HS4, 5′HS3 and 5′HS2 [46] but not as highly as with all four HS [12]. These data suggest that it will be important to compare the utility of the modified intron 2 in 5′HS3 and larger LCR cassette vectors [45] in the in vivo environment of bone marrow transplantation in preclinical models of hemoglobinopathy.

## Methods

### Plasmid Construction and Generation of Transgenic Mice

Intron modifications were performed largely by PCR as described in the Supplementary Methods (Text S1 and Table S1). All introns were reconstructed as *Bam*HI-*Eco*RI fragments that were inserted into the *Bam*HI-*Eco*RI sites flanking intron 2 in BGT64. To generate transgenic mice, the constructs were isolated as *Cla*I fragments and purified using Elutip-d columns (Schleicher and Schuell, Dassell, Germany), ethanol precipitated, resuspended in injection buffer (10 mM Tris-HCl pH 7.5, 0.2 mM EDTA) and diluted to 0.1–0.5 ng/μl. Injected FVB mouse eggs were transferred into recipient CD1 females and fetuses dissected at day 15.5. Fetal heads were collected for identification of transgenic animals by slot or Southern blot hybridization and fetal liver was split in half for subsequent DNA and RNA analysis from samples frozen at −80°C.

### DNA Analysis

Southern transfer and hybridization were by standard procedures. Copy number determination was performed using a Molecular Dynamics PhosphorImager (Sunnyvale, CA). Single copy animals showed a single random-sized end-fragment in *Bam*HI and *Eco*RI digests hybridized with the appropriate globin intron 2 probe. With multicopy animals, the intensity of the end-fragment was defined as 1 copy and was used to calculate the copy number of the multicopy junction-fragment in the same lane. The intactness of the transgene in the DNA sample was verified by Southern blot analysis using *Pst*I digests hybridized to a 5′HS3 or globin intron 2 probe and an endogenous *mTHY-1* probe. Nonintact transgenes were not included in the calculation of copy number. A loading control of bred single copy transgenic mouse DNA included in the *Pst*I Southerns permitted mosaic analysis as described [12]. Mice that were highly mosaic were excluded from study after demonstration that <25% of the fetal liver cells contained intact transgenes.

### Quantitative PCR

We applied real-time SYBR Green PCR and the standard curve method on an ABI 7900 for analysis of lentiviral copy number, and for mosaic analysis in transgenic mice taking into account the transgene copy number deduced by Southern blot analysis. The product amplified using primers specific for the shared 3′ enhancer region of the transgenes was normalized to the amount of product amplified with mouse β-actin primers [42]. For each primer pair (Table 1) a standard curve of 10–10^5^ copies was generated using diluted DNA from BGT50 single copy, bred transgenic mice. Every PCR reaction was performed in duplicate or triplicate in a volume of 20 µl that contained 10 µl of 2× SYBR Green reagent (Applied Biosystems), 0.2 mM of each primer, DNA representing 10^3^–10^5^ copies of target sequence and H_2_O. Cycling conditions were 2 minutes at 50°C, 10 minutes at 94°C, [30 seconds at 94°C, 1 minute at 60°C (actin) or 65°C (3′ enhancer)]×50 cycles. SYBR Green detection was followed by agarose gel electrophoresis and/or Melting Curve analysis using Dissociation Curves software, to ensure that only the target sequence was amplified.

**Table 1:** Real-time PCR primers

3′ Enhancer	41306–41328 F (3′ of Aγ-Exon3)	GGCTGGATTGATTGCAGCTGAGT
	64547–64525 R (β 3′Enh)	GCACCATAAGGGACATGATAAGG
Mouse β-actin NM_007393	694-713 F	AGAGGGAAATCGTGCGTGAC
	831-815 R	CAATAGTGATGACCTGGCCGT
Human hybrid globin U01317	β-5′ UTR 62165-62187 F	CTAGCAACCTCAAACAGACACC
	Aγ-exon 2 39762-39742 R	GTTGCCCATGATGGCAGAGGC
Mouse β-globin major gene J00413.1	3000-3021 F exon2	TGGGTAATGCCAAAGTGAAGGC
	3853-3834 exon3	AATCCTTGCCAAGGTGGTGG
Mouse RNA Pol II (RPB1) U37500.1	1694-1714 F	TCACGGCAGTACGCAAATTCA
	1783-1761 R	CATCCCACGTGGACAGAAACATT

### Quantitative RT-PCR

Quantitative reverse transcriptase SYBR Green PCR was used to estimate Aγ-globin expression. Total RNA was obtained using Trizol reagent (Invitrogen), DNase treated and cDNA synthesized using Superscript II Reverse Transcriptase (Invitrogen). The primer pairs used traverse exon junctions. Human hybrid globin (U01317) primer pair: 62165-62187 F for β-5′UTR, and 39762-39742 R for Aγ-Exon 2. Standard curves were generated using serial dilutions of cDNA from BGT50 single copy, bred transgenic mice or MEL BGT50 cDNA. Aγ-globin expression data were normalized relative to mouse RNA polymerase II large subunit or mouse βmajor-globin. Mouse βmajor-globin (J00413.1) primer pair: 3000-3021F, 3853-3834 R. Mouse RNA polymerase II largest subunit (RPB1) mRNA (U37500.1): 1694-1714 F and 1783-1761 R. Cycling conditions were 2 minutes at 50°C, 10 minutes at 94°C, [30 seconds at 94°C, 1 minute at 69°C (human β/γ-globin), 65°C (Mouse polymerase II or β-globin)]×50 cycles. Specificity of PCR was verified by Melting Curve analysis using Dissociation Curves software.

### Site Specific Integration into MEL Acceptor Cells

MEL acceptor and MEL BGT50 cell lines were described previously [42] and maintained in DMEM supplemented with 10% heat-inactivated fetal calf serum (FCS), L-glutamine 100 µM, penicillin 100 µg/ml and streptomycin 292 µg/ml (Invitrogen). The SFV plasmid is used to deliver LCR β/γ-globin sequences to the genomic FRT site. SFV contains a single FRT site, the Hygromycin resistance (Hygro) gene and several unique restriction sites (*Not*I, *Bam*HI) for cloning. The BGT64, BGT156 and BGT158 transgenes were subcloned by *Eco*RV digestion and A-tailing into pGEM-T Easy, digested with *Not*I and cloned into the unique *Not*I site. Transient transfection of the MEL acceptor cell line was performed with circular plasmids as described previously [42]. In brief: 100 µg SFV based BGT156 or BGT158 vectors were transfected together with 100 µg pEGFP-FLP-C1 (generous gift of D.E. Sabath) by a single pulse at 260 V and capacitance of 960 µF. Cells were plated 48 h after transfection at different densities (10^3^, 10^4^ per 200 µl in 96-well plates) for selection in medium containing 500 µg/ml of Hygromycin (Invitrogen). To identify cell clones with integration into the target FRT site, flow cytometric analysis was performed to identify cells that do not express β-galactosidase (β-gal) activity (FACS-GAL) using fluorescein di-β-D-galactopyranoside (FDG) as described. Flow cytometry was performed on a FACScan (Becton Dickinson) using 488 nm argon laser excitation and a 525 nm bandpass filter to detect FITC fluorescence emission and 620 nm bandpass filter to detect PI fluorescence emission.

### Flow Cytometry for Intracellular Aγ-Globin Protein

Flow cytometric analysis of Aγ-globin expression was performed as described [67],[68]. In brief, 5×10^6^ MEL cells were suspended in 1 mL HBSS with 4% formaldehyde and incubated at room temperature for 30 min. Cells were then permeabilized by serial washes in cold acetone, washed with cold HBSS-2% FBS and stained with a FITC-conjugated anti-human hemoglobin (γ-chain) monoclonal antibody (Cortex Biochem) for 30 min on ice. The cells were washed again with HBSS-2% FBS and analyzed by flow cytometry on a FACScan (BD Biosciences) using CellQuest software.

### 3D DNA FISH

3D DNA FISH preserves the three dimensional structure of the nucleus using a protocol adapted from Solovei et al [69]. Slides were washed with 100% ethanol and 0.1M HCl before coating with Poly-L-ornithine (Sigma). Cells were washed and left to attach on Poly-L-ornithine covered slides for 10 min. The cells were fixed in 4% PFA/PBS for 10 min, quenched with 50 mM NH_4_Cl/PBS for 5 min and permeabilized in 0.5% Triton X-100/PBS for 5 min at RT. Slides were stored in 20% glycerol/PBS overnight at 4°C before repeated freezing in liquid nitrogen. Slides were stored in 50% formamide/2× SSC overnight. Cells were denatured in 70% formamide/2×SSC for 5 min. Probes were labeled with DIG-nick translation mix (Roche), precipitated overnight with mouse Cot-1 DNA and salmon sperm DNA and denatured at 95°C for 10 min before addition to denatured cells. For detection of endogenous mouse β-globin loci, the 189 kb BAC RP23-370E12 was labeled with DIG-nick translation mix (Roche) and detected by anti-DIG-rhodamine (Roche, 1/100 dilution). Probes were hybridized overnight at 37°C. Slides were washed three times in 50% formamide/50% 2×SSC at 42°C and once in 0.5×SSC at 62°C. Cells were then blocked in 1% blocking reagent (Molecular Probes) for 30 min followed by an intermediary HRP-labeling step by sheep anti-DIG-POD (Roche, 1/100 dilution) or rabbit anti-DIG-HRP (DakoCytomation, 1/400 dilution) for 30 min. HRP-conjugated DIG-labeled probes were detected using Alexa Fluor 546-labeled tyramide (Molecular Probes) [43]. Slides were washed in 2× SSC, counterstained in DAPI and mounted in antifade.

### 3D DNA FISH Image Analysis

Images were captured using a Zeiss Axiovert 200M Inverted Microscope equipped with AxioCam HRm (BGT158, BGT156 and MEL acceptor cell experiments), or with Axiovert 200 Inverted Microscope equipped with a Hamamatsu Orca AG CCD camera and spinning disk confocal scan head (endogenous β-globin locus and BGT64 analysis). For each randomly chosen field, z-sections spaced 0.2 to 0.275 µm apart were collected and subsequently deconvolved using Axiovision Rel. 4.6 or Volocity. The z-section containing the FISH signal was analysed to determine the distance of the transgene relative to the nuclear periphery, as defined by a sharp drop in DAPI staining. The distance of the transgene relative to the nuclear periphery was normalized with the radius of the nucleus at that z-section. From 50 to 170 FISH signals were measured for each experiment and each experimental condition was repeated at least two times.

### ESI Fixation and Embedding

Cells were fixed in 2% paraformaldehyde in PBS for 10 min, permeabilized in 0.5% Triton-X 100 buffered in PBS and fixed with 1% glutaraldehyde for 5 min. Cells are rinsed three times with PBS between treatments. The cells were then dehydrated with ethanol in steps of 30%, 50%, 70%, 90% and 100% for 30–90 minutes each. Cells were then embedded in Quetol resin as previously described [70].

### ESI Electron Microscopy

Cells were sectioned to 70 nm in thickness with an ultra microtome onto copper mesh grids before coating with a 3 nm carbon film to stabilize sections. Whole nuclei sections and regions of interest were imaged at 200 kV with a Tecnai 20 (FEI; Eindhoven, The Netherlands) transmission electron microscope equipped with a Gatan imaging filter. To measure cell nuclei size and heterochromatin distribution, phosphorus enhanced mass images were collected at 155 eV with a CCD camera. Element-specific images were obtained as described previously [44]. Phosphorus images were acquired at 120 eV and 155 eV and nitrogen images with energy loss windows at 385 eV and 415 eV. Images were processed using Digital micrograph software and Photoshop.

### ESI Image Analysis

To assess the impact of HMBA induced differentiation of MEL cells on nuclear size low magnification phosphorus enhanced mass images were measured with ImageJ software. Since nuclei were elliptical in shape, both the long and the short axis were measured and averaged, and compared between control and differentiated states. The average diameter of individual axis were calculated, and compared between treatments. To ascertain the impact of reduced nuclear size on chromatin distribution in differentiated cells, ImageJ was used to analyze mass images. The total volume of peripheral heterochromatin was measured and normalized to the length of the perimeter, to account for variations in observed section areas. Qualitative assessments were made of the higher-resolution element specific ratio maps to assess changes in chromatin fiber structure and compaction.

### Lentiviral Vector Production and Infection

The PL.SIN.cHS4 lentivirus vector was created using PCR and multiple cloning steps as described in the Supplementary Methods (Text S1 and Table S1). The LCR β/γ-globin transgenes were isolated as *Cla*I-*Eco*RV fragments and inserted in the antisense orientation into a blunted *Bam*HI and a *Cla*I site in the lentivirus vector. Lentivirus vectors were produced in 293-T cells cultured in Dulbecco's modified Eagle's medium (Invitrogen) with 10% fetal bovine serum (FBS) (Invitrogen). The 293-T cells were plated at a density of 8×10^6^ in T-75 flasks. The following day, the cells were transfected using Lipofectamine 2000 (Invitrogen) with 10 µg human papillomavirus (HPV) 275 (gag/pol expression plasmid), 10 µg P633 (rev expression plasmid), 10 µg HPV17 (tat expression plasmid), 5 µg pHCMV-VSV-G (VSV-G expression plasmid) and 15 µg of PL.SIN.cHS4.BGT plasmid. The lentiviruses were collected in 20 mL media after 48 h, filtered through 0.45 µm filters and concentrated by ultracentrifugation at 4°C, 2 h, 30,000 rpm with T-865 rotor (Sorvall). The viral pellet was resuspended to a final volume of 80 µl Hanks' balanced salt solution (HBSS, Invitrogen) overnight at 4°C. Next day, 2×10^5^ murine erythroleukemia (MEL) cells were infected with lentiviruses at different doses (4, 10 or 40 µl) in the presence of 8 µg/ml polybrene (Sigma). After 24 h, the medium was replaced with fresh growth medium. To analyze expression of the Aγ-globin, infected MEL cells were induced by 5 mM *N, N*'-hexamethylene-bisacetamide (HMBA, Aldrich) for 6 days. Virus titers were estimated by flow cytometry using the formula: NxM/100V, where N is the number of MEL cells used for infection, M is % expressing cells, and V is the volume of concentrated virus used for infection (ml).

## Supporting Information

Figure S1 The transgene is most frequently proximal to heterochromatin before induction in MEL BGT158 cells. The distance of the transgene signal from the nearest DAPI-rich heterochromatin was measured in a z-section. Proximity is arbitrarily defined as being within 1 µm of heterochromatin.
(0.55 MB TIF)

Figure S2 Characterization of MEL cell induction using surface markers of erythroid development. Uninduced and 3 day HMBA treated MEL cells were stained with the following antibodies to detect early (CD117), intermediate (Ery1 and CD71) and late stages (Ter119) of erythroid development. Positive controls include E12.5 mouse fetal liver and Primary Bone Marrow Mastocytes. **Method:** MEL cells were washed with cold HBSS-2% FBS and stained with a PE-conjugated anti-CD117 (BD Pharmingen Cat. #553355) provided by D. Barber or FITC-conjugated anti-CD71 (BD Pharmingen Cat. #553266), or PE-conjugated anti-Ter119 (BD Pharmingen Cat. #553673) for 30 min on ice, or anti-Ery1 antibody (Bacon and Sytkowski 1987, *Blood* 69:103-108) which was followed by an Alexa Fluor 488 goat anti-rat IgG (Molecular Probes A-11006). The cells were washed again with HBSS-2% FBS and analyzed by flow cytometry on a FACScan (BD Biosciences) using CellQuest software. Primary Bone Marrow Mastocytes (BMMC) cells provided by S. Berger were used as positive controls for anti-CD117 antibody and were maintained in Opti-MEM supplemented with 5% FBS, 6% WEHI conditioned medium containing IL-3 and 55 µM beta-mercaptoethanol. As positive controls for anti-CD71, anti-Ter119 and Ery1 antibodies, we used freshly isolated mouse fetal liver cells from E12.5 embryos.
(1.12 MB TIF)

Figure S3 The cHS4 dimer core recombines into a monomer after lentivirus transfer. PCR amplification using primers that overlap the insulator-LTR junctions demonstrates presence of a monomer cHS4 core element. *LTR* **cHS4** Forward cHS4 primer 5′-*TCCCAAAGAAGACAAGAT* **GTCG** *LTR* **cHS4** Reverse cHS4 primer 5′-*GTACAGGCAAAAAGCAG* **GTCGAAGC**

(5.44 MB TIF)

Table S1 List of primer sequences used in PCR reactions for construction of LCR β/γ-globin transgenes and lentiviral vectors.
(0.03 MB DOC)

Text S1 Supplementarty methods
(0.04 MB DOC)

Video S1 Example 3D DNA FISH reconstruction of transgene localization in uninduced MEL BGT158 cells. The transgene is detected as a red signal by a 10 kb DIG labeled probe using Alexa Fluor 546 labeled tyramide signal amplification with DAPI counterstained nuclear DNA in green. DAPI-rich areas correspond to heterochromatin.
(5.06 MB MOV)
