# Supplementary figures and images for: β-Globin LCR and Intron Elements Cooperate and Direct Spatial Reorganization for Gene Therapy
Source: PLoS Genet. 2008 Apr 18;4(4):e1000051. doi: 10.1371/journal.pgen.1000051 (PMC2271131; doi:10.1371/journal.pgen.1000051)

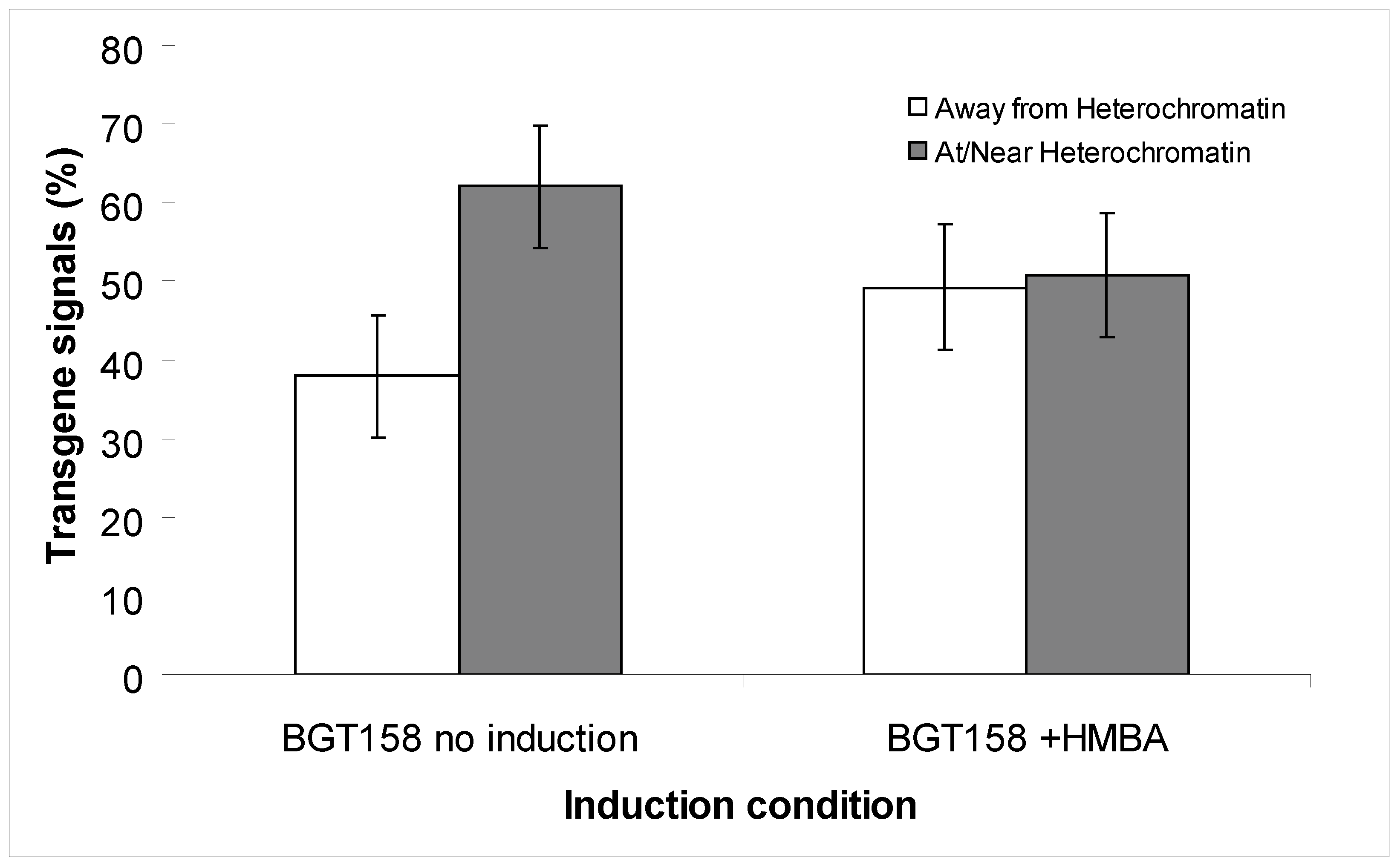

Supplement: Figure S1 — The transgene is most frequently proximal to heterochromatin before induction in MEL BGT158 cells. The distance of the transgene signal from the nearest DAPI-rich heterochromatin was measured in a z-section. Proximity is arbitrarily defined as being within 1 µm of heterochromatin. (0.55 MB TIF) [file pgen.1000051.s001.tif]

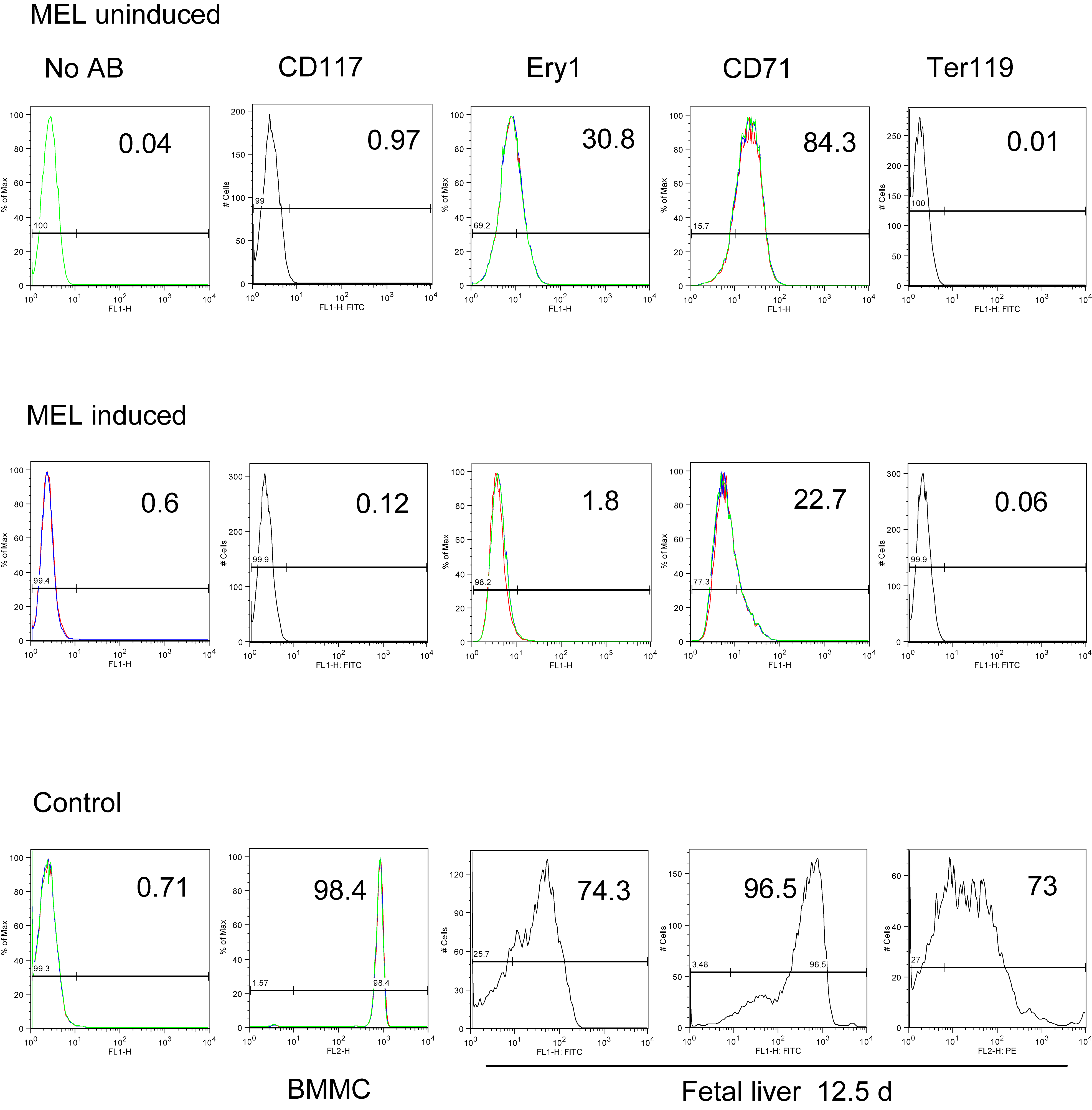

Supplement: Figure S2 — Characterization of MEL cell induction using surface markers of erythroid development. Uninduced and 3 day HMBA treated MEL cells were stained with the following antibodies to detect early (CD117), intermediate (Ery1 and CD71) and late stages (Ter119) of erythroid development. Positive controls include E12.5 mouse fetal liver and Primary Bone Marrow Mastocytes. Method: MEL cells were washed with cold HBSS-2% FBS and stained with a PE-conjugated anti-CD117 (BD Pharmingen Cat. #553355) provided by D. Barber or FITC-conjugated anti-CD71 (BD Pharmingen Cat. #553266), or PE-conjugated anti-Ter119 (BD Pharmingen Cat. #553673) for 30 min on ice, or anti-Ery1 antibody (Bacon and Sytkowski 1987, Blood 69:103-108) which was followed by an Alexa Fluor 488 goat anti-rat IgG (Molecular Probes A-11006). The cells were washed again with HBSS-2% FBS and analyzed by flow cytometry on a FACScan (BD Biosciences) using CellQuest software. Primary Bone Marrow Mastocytes (BMMC) cells provided by S. Berger were used as positive controls for anti-CD117 antibody and were maintained in Opti-MEM supplemented with 5% FBS, 6% WEHI conditioned medium containing IL-3 and 55 µM beta-mercaptoethanol. As positive controls for anti-CD71, anti-Ter119 and Ery1 antibodies, we used freshly isolated mouse fetal liver cells from E12.5 embryos. (1.12 MB TIF) [file pgen.1000051.s002.tif]

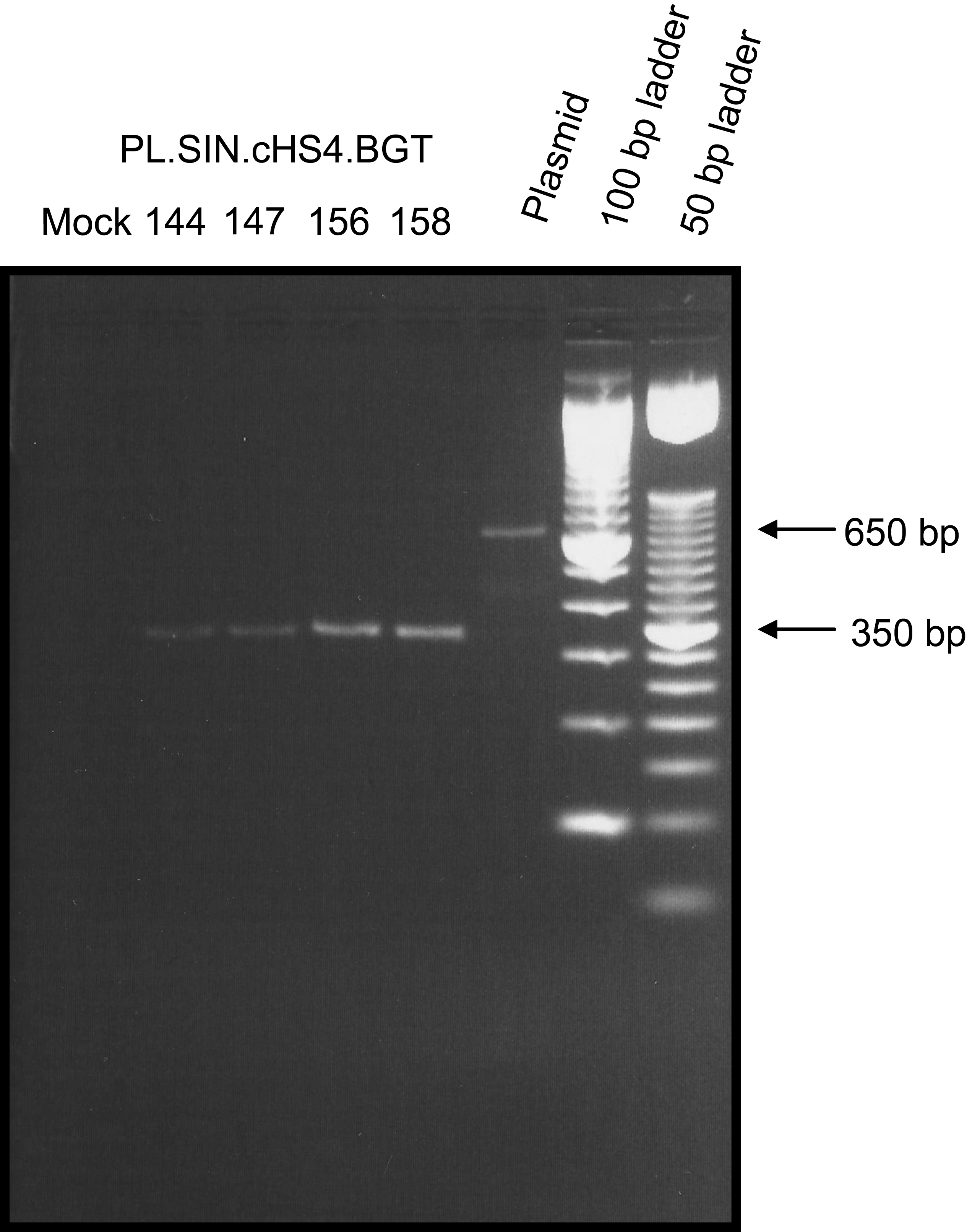

Supplement: Figure S3 — The cHS4 dimer core recombines into a monomer after lentivirus transfer. PCR amplification using primers that overlap the insulator-LTR junctions demonstrates presence of a monomer cHS4 core element. LTR cHS4 Forward cHS4 primer 5′-TCCCAAAGAAGACAAGAT GTCG LTR cHS4 Reverse cHS4 primer 5′-GTACAGGCAAAAAGCAG GTCGAAGC (5.44 MB TIF) [file pgen.1000051.s003.tif]
