# Supplementary material for: β-Globin LCR and Intron Elements Cooperate and Direct Spatial Reorganization for Gene Therapy
Source: PLoS Genet. 2008 Apr 18;4(4):e1000051. doi: 10.1371/journal.pgen.1000051 (PMC2271131; doi:10.1371/journal.pgen.1000051)
Supplement: Table S1 — List of primer sequences used in PCR reactions for construction of LCR β/γ-globin transgenes and lentiviral vectors. (0.03 MB DOC) [file pgen.1000051.s004.doc]

| Table S1: List of primer sequences used in PCR reactions for construction of  LCR β/γ-globin transgenes and lentiviral vectors Primers Primer Sequence Function | | | |
| --- | --- | --- | --- |
| **BGT144** | 5'γizedβIVS2 (S) | 5' GCTGCATGTGGATCCTGAGAACTTCA**A**GGTGAGTCTATGGGACC 3' | Arg→Lys |
|  | B64 Oct (AS) | 5' GGTCAATAT**ATGCAAAT**TGTTACTTCTCCC 3' | Oct-1 |
|  | B64 Oct (S) | 5' GGGAGAAGTAACA**ATTTGCAT**ATATTGACC 3' | Oct-1 |
|  | 3'γizedβIVS2 (AS) | 5' TCAGGGGTGAATTCTTTGCCAAAGTGA**AT**GGCCAGCACA**GT**GACCAGCACG 3' | Cys→Thr  His→Iso |
| **BGT145** | B54 (S) | 5' CTTTGCCCAGCTGAGTGAACTGC 3' |  |
|  | B54Oct (AS) | 5' GCGCCAATATATGC**ATGCAAAT**TGCATCTTTTTAACGACC 3' | Oct-1 |
|  | B54Oct (S) | 5' GGTCGTTAAAAAGATGCA**ATTTGCAT**GGATATATTGGCTC 3' | Oct-1 |
|  | 3'βIVS2/E3 (AS) | 5' TCAGGGGTGAATTCTTTGCCG 3' |  |
| **BGT 147** | B54 (S) | 5' CTTTGCCCAGCTGAGTGAACTGC 3' |  |
|  | B54 DraI  (AS) | 5' CCCTGGACATACTTT**TTAAA**CATCTGG 3' | *Dra*I |
| **BGT158** | 5'BmgBIclone | 5' GCATGTGGATCCTGAGAACTTCAAGGTGAG 3' |  |
|  | 5'BmgBI | 5' GCTTTCTTCTTTT**CACGTC**CTTAAGTGTTTATCTTATTTC 3' | *Bmg*BI |
|  | 3'BmgBI | 5' GAAATAAGATAAACACTTAAG**GACGTG**AAAAGAAGAAAGC 3' | *Bmg*BI |
|  | 3'BmgBIclone | 5' CTCAGGGGTGAATTCTTTGCCAAAGTGAATG 3' |  |
|  | 5'IgμMAR | 5' GGGTAATTCATTTTCAAAATTAGGTTATGTAAG 3' | IgμMAR |
|  | 3'IgμMAR | 5' GATTTTATGCGCCTGCGTCGGTACTAG 3' | IgμMAR |
| **PL.sin.sal** | PLsal/sinA (S) | 5' AAGACCCCAACGAGAAGCGCG 3' |  |
|  | PLsal/sinB (AS) | 5' **GTCGAC**ATCTTGTCTTCTTTGGGAGTG 3' | *Sal*I |
|  | PLsal/sinC (S) | 5' **GTCGAC**CTGCTTTTTGCCTGTACTGGG 3' | *Sal*I |
|  | PLsal/sinD (AS) | 5' GTTGTGTGGAATTGTGAGCGG 3' |  |
