# Supplementary material for: β-Globin LCR and Intron Elements Cooperate and Direct Spatial Reorganization for Gene Therapy
Source: PLoS Genet. 2008 Apr 18;4(4):e1000051. doi: 10.1371/journal.pgen.1000051 (PMC2271131; doi:10.1371/journal.pgen.1000051)
Supplement: Text S1 — Supplementarty methods (0.04 MB DOC) [file pgen.1000051.s005.doc]

**Text S1 - SUPPLEMENTARY METHODS**

**A. Construction of LCR β/γ-globin transgenes with modified introns**

**BGT144** and **BGT145** plasmids were created on the basis of BGT64 that contains the β-globin intron 2 (βIVS2) with a deletion of the 372bp AT-rich (ATR) region. Two-step PCR-based site-specific mutagenesis was used to introduce an Oct-1 site into βIVS2 and also change three codons from β-globin to Aγ-globin sequence. The 3 remaining β-globin codons were a result of the previous cloning of βIVS2 between Aγ-globin exon 2 and 3 at the restriction sites *Bam*HI and *Eco*RI in each exon. The first PCR reaction used the 5'γized βivs2 (S) primer (Table S1) that covers the 5’ intron junction, and changes the β-globin codon arginine (position 56), to the Aγ-globin codon lysine and antisense Oct-1(AS) containing primer. A concurrent reaction used 3'γized βivs2 (AS) in order to change two β-globin codons, cysteine (position 556) and histidine (position 568) to Aγ-globin codons threonine and isoleucine respectively and Oct-1 (S) sense primer. The products from both reactions were combined, annealed at the 30 bp sequence that contains the Oct-1 consensus and used as a template for the second step PCR with 5'γized βivs2 primer (S) and 3'γized βivs2 (AS). The same two-step PCR-based site-specific mutagenesis approach was used to introduce the Oct-1 site into Aγ-globin intron 2 to create the BGT145 construct. In this case, BGT54 which has Aγ-globin intron 2 and encodes completely wild-type Aγ-globin protein, served as a template for PCR. Primers used are listed in Table S1. BGT144 and BGT145 intron 2 PCR products were subcloned into pGEM-T and verified by sequencing. To create the final LCR β/γ-globin transgenes, the BGT144 and BGT145 intron 2 sequences were released by *Eco*RI and *Bam*HI digestion, and ligated between the *Eco*RI and *Bam*HI sites of BGT64.

**BGT147** has a hybrid intron 2 that contains the 5' Aγ-globin region, Oct-1 site and 3' β-globin region created in several steps. First, the 5' Aγ intron 2 was PCR amplified using B54 (S) and B54 Dra (AS) primers (Table S1) using BGT54 DNA as template. B54 Dra (AS) primer was used to create a *Dra*I site within intron 2. The PCR product was digested with *Bam*HI and *Dra*I, subcloned into pGEM-T and confirmed by sequencing. Second, the 5' Aγ-globin region of intron 2 was released from pGEM-T by digestion with *Bam*HI and *Dra*I. The 3' β-globin intron 2 was isolated from BGT144 with *Dra*I and *Eco*RI. The 5' Aγ-globin and 3' β-globin regions were ligated together into pLitmus 38. Subsequent *Bam*HI and *Mlu*NI digestion of pLitmus 38 containing the new hybrid intron 2 released the 5' Aγ-globin region that was exchanged for the *Bam*HI-*Mlu*NI fragment from BGT145 that contains the 5' Aγ-globin intron 2 with the Oct-1 site. Third, this new BGT147 hybrid intron 2 was released by digestion with *Bam*HI and *Eco*RI and cloned between the *Bam*HI and *Eco*RI sites of BGT64. BGT147 encodes fully wild-type Aγ-globin.

**BGT156** was created by ligating the Igμ 3'MAR (gift from D. Kohn and M. Shulman) into intron 2 sequence from BGT64. A pGEM plasmid containing the *Bam*HI-*Eco*RI fragment of BGT64 intron 2 was partially digested with *Rsa*I to yield a linearized 3.7 kb fragment and dephosphorylated. The 309 bp *Xba*I/*Eco*RI fragment containing Igμ 3'MAR was digested, blunt ended and ligated into the linearized 3.7 kb BGT64 intron 2 backbone. This intron 2 containing the Igμ 3'MAR was excised as an 856 bp *Bam*HI-*Eco*RI fragment and was cloned between the *Bam*HI-*Eco*RI sites of BGT50. The BGT156 intron 2 sequence was verified. The BGT156 transgene does not encode fully wild-type Aγ-globin protein because it retains the 3 codon modifications present in BGT64.

**BGT158** intron 2 contains the Oct-1 site, Igμ 3'MAR and intronic enhancer and was generated in several steps. First, two-step PCR-based site-specific mutagenesis was used to introduce a *Bmg*BI restriction site downstream of the Oct-1 site in the BGT144 intron 2. PCR product was subcloned into pGEM-T and sequence verified. Second, Igμ 3'MAR sequence was PCR amplified from BGT156 intron 2, subcloned into pGEM-T and sequenced. Sequences of primers used to clone BGT158 are indicated in the Table S1. The *Ec*oRI Igμ 3'MAR fragment was blunted and cloned into the *Bmg*BI site of intron 2 in the context of pGEM-T. Finally, this BGT158 intron 2 was *Bam*HI and *Eco*RI digested and inserted between the *Bam*HI and *Eco*RI sites of BGT144. BGT158 encodes fully wild-type Aγ-globin.

**B. Construction of the PL.SIN.cHS4 lentivirus vector**

The original lentivirus (HPV436 renamed here PL.wt.β87globin) was received from P. Leboulch and contains wild-type HIV-1 LTRs, a Rev-response Element (RRE), a central polypurine tract (cppt) and expression of viral RNA is driven by the Tat-activated region (TAR) [1]. The construct also contained in reverse orientation the β87-globin gene with a mutation that encodes an anti-sickling protein under the control of the LCR elements 5'HS2-4 and the β-globin promoter. To create an insulated self-inactivating version of this lentivirus vector bearing the LCR β/γ-globin transgenes required multiple steps.

**PL.SIN.EF1αEGFP**

First, the 3'LTR was subcloned as a *Kpn*I-*Eco*RI fragment to create pGEM-LTR, which has a *Bam*HI site in the polylinker adjacent to KpnI. The SIN deletion was made by digesting this plasmid with *Eco*RV and *Pvu*II and blunt end religating at these sites to make pGEM-SINLTR. Second, a full length lentivirus vector was reconstructed by replacing the original β87globin cassette and wild-type 3'LTR (present on a *Bam*HI-*Eco*RI fragment in PL.wt.β87globin) with the *Bam*HI-*Eco*RI SIN LTR fragment. This PL.SIN.X plasmid has a polylinker with unique *Bam*HI, *Cla*I and *Kpn*I sites upstream of the SIN 3'LTR but no transgene. Third, an EF1α-EGFP SIN lentivirus was made by digesting this transcription unit from the KA436 retrovirus vector [2] using *Hpa*I and adding *Bam*HI linkers before cleaving the 3' end with *Cla*I. This *Bam*HI-*Cla*I fragment was inserted into the *Bam*HI-*Cla*I polylinker sites in PL.SIN.X to create PL.SIN.EF1αEGFP.

**PL.SIN.cHS4.EF1αEGFP**

Fourth, to insert the dimer core cHS4 insulator into the SIN 3'LTR it was necessary to create a *Sal*I site at the SIN deletion using PCR. The upstream and downstream parts of the SIN 3'LTR were amplified using primers that contain a *Sal*I site at the SIN deletion, and the two products cloned into pGEM and sequenced. These were then purified as an upstream *Kpn*1-*Sal*I fragment and a downstream *Sal*I-*Eco*RI fragment that were ligated together and used to replace the *Kpn*I-*Eco*RI SIN 3'LTR in PL.SIN.EF1αEGFP. This new PL.sal.EF1α-EGFP construct thus contains a 3'LTR with a unique *Sal*I site at the SIN deletion. To insert the cHS4 dimer core fragment into this *Sal*I site, the pNi-CD plasmid (kindly provided by G. Felsenfeld) was digested in its flanking polylinker with *Eco*RI and *Kpn*I before blunting with klenow. This blunt fragment was inserted in either the reverse (A) or forward (B) orientations into the blunted *Sal*I site of PL.sal.EF1αEGFP to create PL.SIN.cHS4.EF1αEGFP.

**PL.SIN.cHS4.BGT lentivirus vector series**

Fifth, the B orientation of the insulated SIN lentivirus vector was used as the backbone for the 5'HS3 β/γ-globin transgenes. The BGT144, 147, 156 and 158 transgenes were released as *Cla*I-*Eco*RV fragments and inserted in the antisense orientation into a blunted *Bam*HI and *Cla*I site after removing the EF1α-EGFP cassette. To generate the LCR (5'HS4, 5'HS3, 5'HS2) β/γ-globin BGT161 vector, the 3.0 kb *Not*1-*Sal*1 LCR fragment from BGT14 was used to replace the blunted *Cla*1-*Sal*1 5'HS3 fragment in the PL.SIN.cHS4.BGT158 transgene. The BGT159 and BGT160 vectors were obtained by ligation of the *Bam*HI-*Pin*AI fragment of intron 2 from BGT50 or BGT64 respectively into the *Bam*HI-*Pin*AI site of BGT161.

**REFERENCES**

1. Pawliuk R, Westerman KA, Fabry ME, Payen E, Tighe R, et al. (2001) Correction of sickle cell disease in transgenic mouse models by gene therapy. Science 294: 2368-2371.

2. Dalle B, Rubin JE, Alkan O, Sukonnik T, Pasceri P, et al. (2005) eGFP reporter genes silence LCRbeta-globin transgene expression via CpG dinucleotides. Mol Ther 11: 591-599.
